# Epidemiological features of influenza circulation in swine populations: A systematic review and meta-analysis

**DOI:** 10.1371/journal.pone.0179044

**Published:** 2017-06-07

**Authors:** Eugénie Baudon, Marisa Peyre, Malik Peiris, Benjamin John Cowling

**Affiliations:** 1 WHO Collaborating Centre for Infectious Disease Epidemiology and Control, School of Public Health, The University of Hong Kong, Hong Kong Special Administrative Region, China; 2 Animal and Integrated Risk Management Research Unit (AGIRs), French Agricultural Research Center for International Development (CIRAD), Montpellier, France; University of Georgia, UNITED STATES

## Abstract

**Background:**

The emergence of the 2009 influenza pandemic virus with a swine origin stressed the importance of improving influenza surveillance in swine populations. The objectives of this systematic review and meta-analysis were to describe epidemiological features of swine influenza (SI) across the world and identify factors impacting swine influenza virus surveillance.

**Methods:**

The systematic review followed the PRISMA guidelines. Articles published after 1990 containing data on SI on pig and herd-level seroprevalence, isolation and detection rates, and risk factors were included. Meta-regression analyses using seroprevalence and virological rates were performed.

**Results:**

A total of 217 articles were included. Low avian influenza (AI) seroprevalence (means pig = 4.1%; herd = 15%) was found, showing that AIV do not readily establish themselves in swine while SIV seroprevalence was usually high across continents (influenza A means pig = 32.6–87.8%; herd = 29.3–100%). Higher pig density and number of pigs per farm were shown by the meta-regression analyses and/or the risk factor articles to be associated with higher SI seroprevalence. Lower seroprevalence levels were observed for countries with low-to-medium GDP. These results suggest that larger industrial farms could be more at risk of SIV circulation. Sampling swine with influenza-like illness (ILI) was positively associated with higher isolation rates; most studies in Europe, Latin and North America were targeting swine with ILI.

**Conclusions:**

To improve understanding of SI epidemiology, standardization of the design and reporting of SI epidemiological studies is desirable. Performance of SI surveillance systems in low-to-medium GDP countries should be evaluated to rule out technical issues linked to lower observed SIV prevalence. Targeting certain swine age groups, farming systems and swine with ILI may improve the surveillance cost-effectiveness. However, focusing on pigs with ILI may bias virus detection against strains less virulent for swine but which may be important as pandemic threats.

## Introduction

The need for improved influenza surveillance in swine for pandemic preparedness was highlighted by the emergence of the pandemic H1N1 virus in 2009 (H1N1pdm09) that spread in humans over the world and which had genetic origins from swine influenza viruses [1]. The H1N1pdm09 virus is a reassortant between swine viruses of the North American triple reassortant and Eurasian-avian lineages [1]. Since then, recurrent swine influenza virus (SIV) spillovers from humans to swine, known as reverse zoonosis events, were observed in all continents, along with H1N1pdm09 virus spreading in the swine population and reassorting with enzootic SIV [2–5]. Even countries such as Australia and Norway, previously considered free from influenza in swine, reported the detection of H1N1pdm09 in swine [6–8]. Recent zoonotic transmissions of a swine H3N2 virus containing the matrix gene from H1N1pdm09 have been detected in the USA. The virus was first isolated in swine in 2010; it was then detected in humans in 2011, referred to as H3N2 variant (H3N2v), and was associated with exposure to pigs at agricultural fairs [9]. No sustained transmission in the human population was detected, although limited human to human transmission was reported.

The enzootic circulation of virus lineages of H1N1, H1N2, and H3N2 subtypes in swine varies by continent and now includes reassortants with the H1N1pdm09 virus [2]. Sporadic introduction of human and avian viruses have been reported in swine. H3N2 and H1N1 human viruses are commonly detected in pigs; some major variants became established and are the main lineages found among enzootic swine viruses. However, apart from those, most human viruses infecting the swine population sporadically fail to circulate long term in pigs [10–12]. H3N1 viruses were also detected in Asia, Europe, North America and Latin America, and were reassortants of local SIV or of human-like viruses and SIV [13–18]. Avian viruses or avian reassortants have been occasionally isolated from pigs with H5N1 and H9N2 subtypes being detected in Asia, and other subtypes including H1N1, H3N2, H5N2, H6N6, H7N2, H4N8 and H11N6 subtypes being detected in different parts of the world [10, 14, 19, 20]. Other infections by avian viruses were reported with a H2N3 reassortant [21] and a H4N6 virus in North America [22]. Viruses from other origins were also detected sporadically, such as an equine H3N8 in China [23], and a H1N7 of human and equine origins in Great Britain [24].

Knowledge of the epidemiology of swine influenza is needed for the development of cost-effective surveillance strategies and of control strategies to limit the spread of these viruses in swine. Swine influenza (SI) epidemiology varies across and within countries due to factors such as climate, pig population and farming practices. Most recent reviews on swine influenza focused on the genetic evolution of the strains circulating in Europe [11, 25], North America [12], Asia [14, 26] and in several continents [2]. A few reviews focused on prevalence, risk factors and isolation rates but they were limited to a geographic region [27] or to a country [28]. This systematic review and meta-analysis aimed to describe epidemiological features of SI to improve the understanding of SI circulation patterns and to better inform surveillance and control strategies. The first objective was to describe epidemiological characteristics of influenza in swine in different countries and factors having an impact on its circulation. Thus, articles with seroprevalence data and risk factor analysis were analyzed in order to describe the extent of SIV circulation in pig populations in the world and to test for risk factors in a meta-analysis. The second objective was to identify factors affecting virus isolation; articles presenting virus isolation rates were reviewed, giving the proportion of viruses successfully isolated, as these data were useful to identify potential factors increasing the probability of isolating viruses.

## Material and methods

### Search strategy

This systematic review followed the PRISMA-P 2015 guidelines (S1 Checklist) [29]. Articles were searched on PubMed, Web of Science, Science Direct, and Scopus using terms related to swine and to influenza (S1 Protocol). The search was performed on 13 October 2014 and updated on 18 January 2016, and the articles published on or after 1990 were included. The references were downloaded and a database of relevant articles was generated.

### Study selection

Article titles and abstracts were screened by two researchers independently to exclude irrelevant articles. The full texts of the remaining articles were then reviewed. The eligibility criteria were as follows: Full text of articles in English, Chinese, Japanese, French and Spanish were kept in the reviewing process. All study designs were eligible. Only articles with original data on influenza circulation in “swine in field conditions” were included, i.e. swine within the production system such as in farms, slaughterhouses, agricultural fairs etc. as opposed to experimental conditions. Selected studies needed to include at least one of the following pieces of data: pig-level or herd-level seroprevalence, virus isolation or detection rate, and risk factors for influenza circulation (S1 Protocol). Experimental studies were excluded, along with studies on the development of new diagnosis methods, vaccines, phylogenetic or antigenic analyses of swine influenza viruses without any epidemiological data mentioned above. Studies on wild boars were not included. The articles published on or after 1990 were kept, except when the studies reported in these articles were entirely performed before 1990.

### Data extraction

The phylogeny of influenza subtypes and strains has been thoroughly reviewed elsewhere for different continents [2, 11, 14, 18, 26, 30] and here we focused on the broader epidemiology of swine influenza. General data on influenza A regardless of subtypes were extracted when available, otherwise detailed information for the investigated subtypes were used (S1 Protocol). In virological studies, isolation and detection rates were differentiated with isolation defined as the successful culture of live virus (e.g. by isolation on MDCK cells or embryonated eggs) and detection defined as detection of SIV with techniques such as RT-PCR in the absence of isolation of the live virus or as a screening method (S1 Protocol). Relevant data were extracted and included pig-level and herd-level seroprevalence, isolation and/or detection rate and related study information including the country, year of start and end of the study, type of study, premise (farm, slaughterhouse, other), premise category (industrial, familial), type of production, ILI symptoms, category or age of pigs, vaccination status etc. Data for risk factor studies were entered in a separate database; when available, only results from multivariate analysis and variables with p-value ≤0.05 were included.

### Data analysis

Data were analyzed in R version 3.2.1 [31]. A general description was done first of the overall search results and then for each data category, i.e. pig-level and herd-level seroprevalence, isolation/detection rate and risk factors. Different study designs with different values were sometimes found in a same article. In this case, each study was considered individually in the analyses. For seroprevalence, a description was done of prevalence data in diseased population (diseased pigs selected partially or in totality for sampling) and general population (sampling on the general population without targeting sick animals specifically), and for the different categories of virus subtypes and strains detected (H1 and H3 of human or swine origin, other human strains, avian strains). For isolation/detection rates, data for studies focusing on outbreaks were compared to data from other studies according to the presence or absence of influenza-like illness (ILI) symptoms (S1 Protocol).

Then meta-regression analyses using mixed-effects models were conducted using the Metafor package [32]. Studies targeting diseased pig populations for seroprevalence and studies on outbreaks for isolation/detection rate were excluded for these analyses. For the seroprevalence, only the results for swine or human H1 or H3 viruses, or influenza A in general were included in the meta-regression models. The data on seroprevalence were heterogeneous with some studies having overall influenza A or aggregated H1 and H3 prevalence data (‘A,H1+H3’), other studies having non-aggregated data on both H1 and H3 (‘H1&H3’), and others only having data on one subtype (‘H1|H3’). Therefore three meta-regressions (M1-3) were conducted retaining only one seroprevalence value per study when an overall prevalence (‘A,H1+H3’) was not available. In M1, the highest seroprevalence value in the study was used; this assumed a total cross-reactivity between the different strains or subtypes. In M2, all the values for one study were added to each other with a maximum of 100% prevalence; this assumed no cross-reactivity. In M3, the same approach as in M2 was used, but studies giving results only for one subtype (‘H1|H3’) were excluded. For virological results, two meta-regression analyses were performed using isolation and detection rates respectively. Variables specific to each study were included when they were reported in most studies together with country specific variables using 2013 data from FAOSTAT [33] and the World Bank [34] (S1 Protocol). Only variables with p-value ≤0.2 in the univariate analysis were included in the final models. For the risk factor studies, the risk factors were divided into categories and a general description was made for each category.

## Results

### Search results

**Fig 1:**
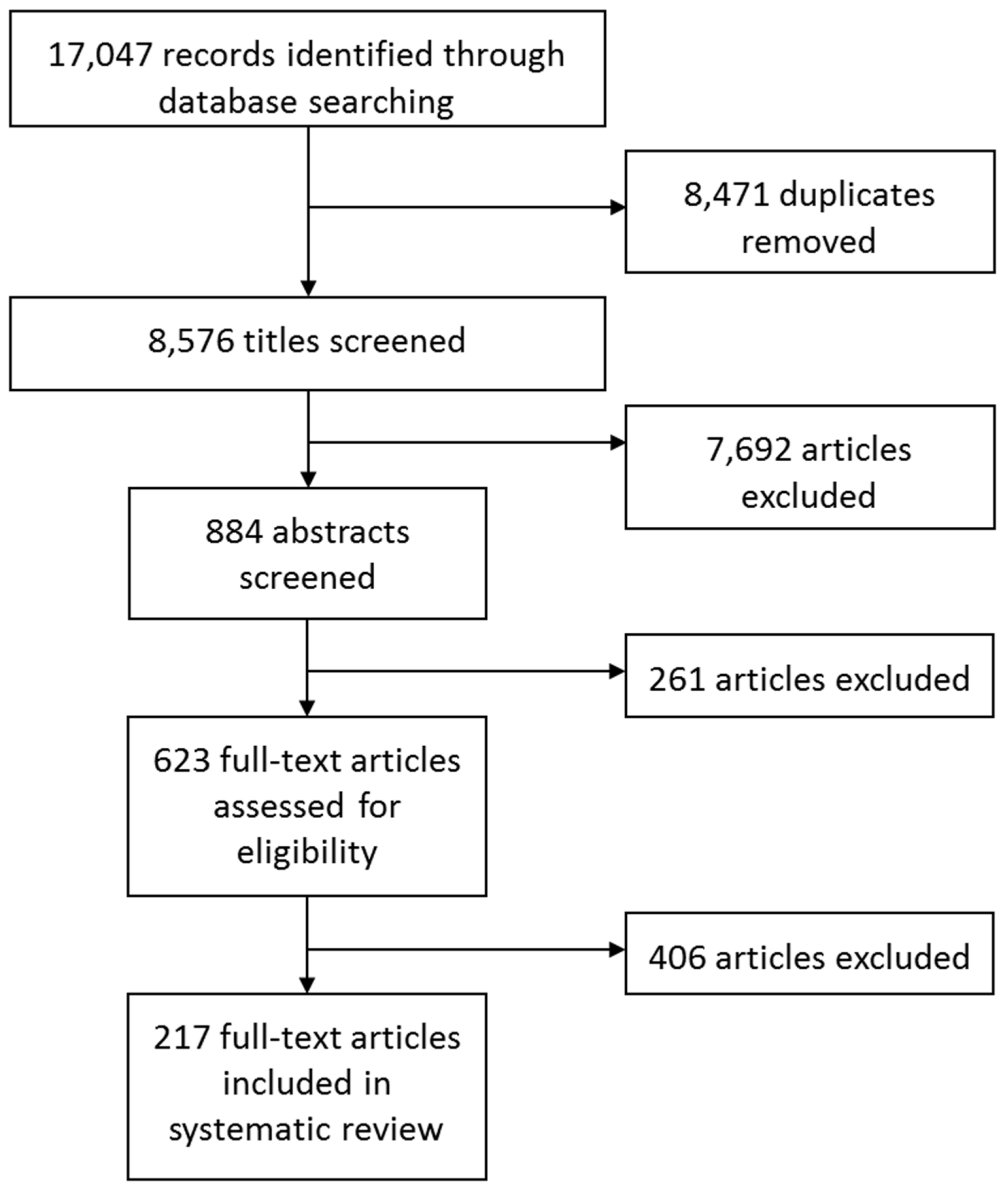
Flow diagram for article selection.

**Fig 2:**
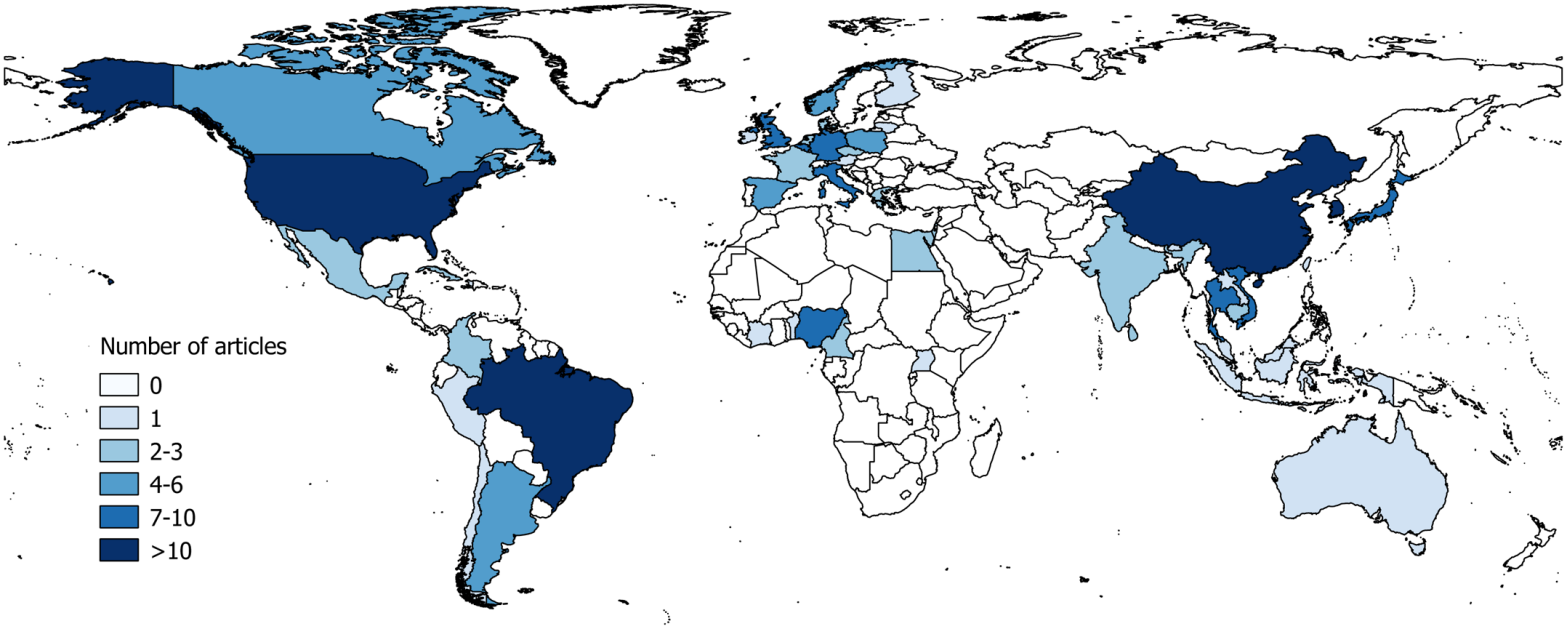
Map of the number of included articles by country. N = 217 articles overall; some articles reported data from several countries.

**Fig 3:**
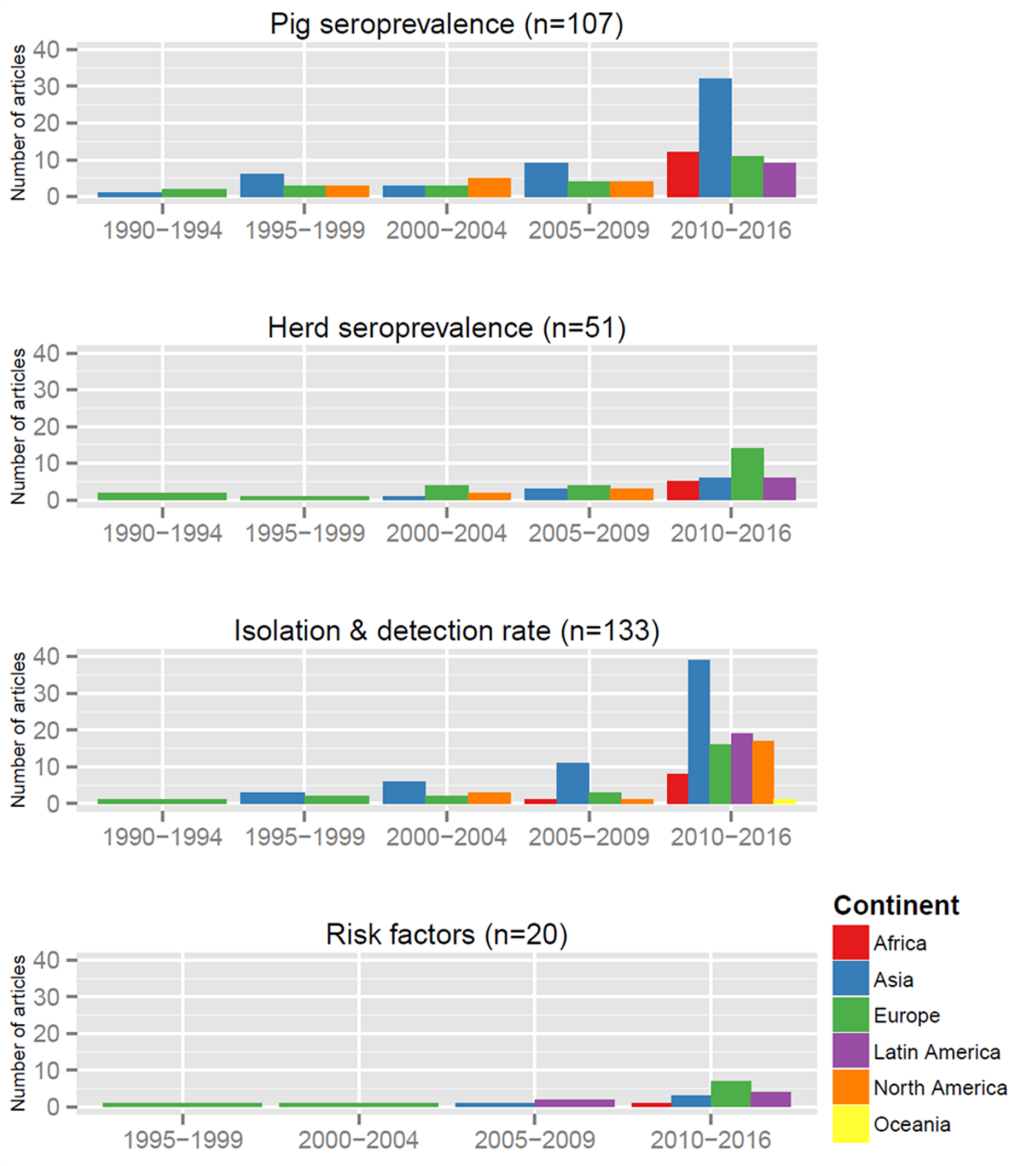
Distribution by year, continent and data type of the included articles. n = number of articles (some articles had data for several categories). N = 217 articles overall.

Of 8,576 articles retrieved from online databases on or after 1990, 623 full-text articles were selected for screening. A total of 217 articles were finally included in the systematic review (Fig 1; S1 References). Overall these articles reported data from 49 countries in six continents (Fig 2). The highest number of articles was for studies carried out in Asia (42.9%) with 13 countries or territories, secondly in Europe (23.5%, 17 countries), North America (14.7%, 2 countries) and Latin America (11.1%, 8 countries), and finally in Africa (7.4%, 8 countries) and Oceania (0.5%, 1 country). More than 10 articles were found for Brazil (n = 11), South Korea (n = 16), the USA (n = 26) and China (n = 42). A total of 107 and 51 articles were included for pig and herd-level seroprevalence data respectively (S2 and S3 Tables), 133 for isolation/detection rates (S4 Table), and 20 for risk factors (S1 Table; Fig 3). There was a clear increase in the number of articles published after the 2009 H1N1 pandemic. For Africa and Latin America, there were no articles published prior to 2009 and 2010 respectively. Prior to 2009, the next pandemic was expected to originate from avian viruses from Asia but instead the emergence of the H1N1 of swine origin in Latin America in 2009 stressed the importance of surveillance in all continents. No seroprevalence or risk factor studies were available in recent years for North America. This was probably due to the important use of vaccination in this region which limits the value of serological investigations.

### Pig-level seroprevalence

#### General description

**Fig 4:**
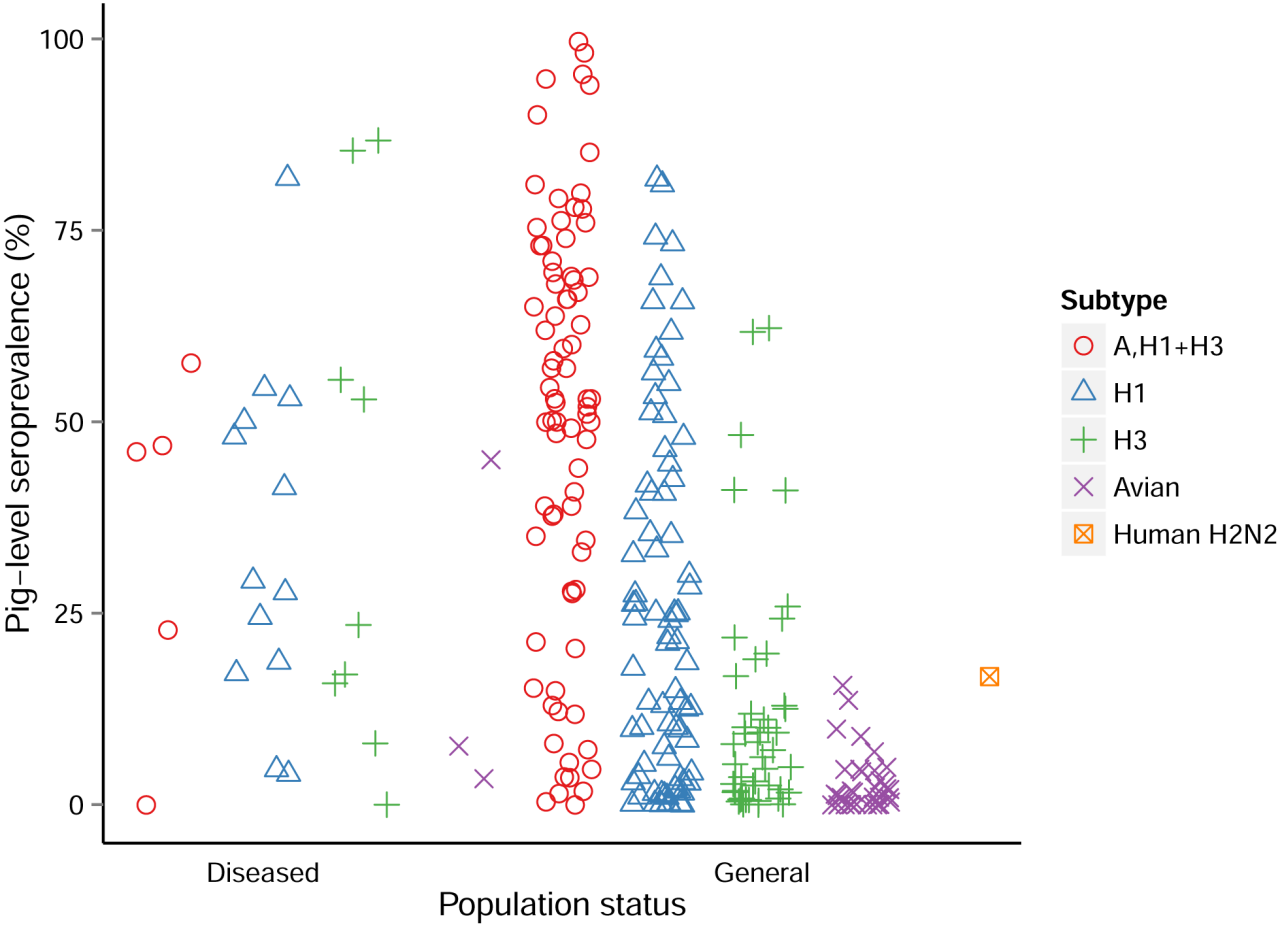
Pig-level seroprevalence distribution by health status and subtypes. N = 164 studies, 271 entries.

A total of 164 studies with pig-level seroprevalence data from 43 countries were retrieved and reported 271 influenza prevalence values. The pig-level seroprevalence values ranged from 0% to 86.7% for diseased populations (16 studies and 30 seroprevalence values) and to 99.7% for general populations (Fig 4); however on average, seroprevalence values were higher for diseased populations (Mean = 34.3%, Median = 28.5%) compared to the general population (Mean = 27.5%, Median = 15.2%). In five studies on the general population, vaccination against SI was reported. In three of these studies, pigs that were vaccinated were excluded [35, 36] or only a very low percentage of the herds (4%) had been vaccinated [37], while in two studies the effect of vaccination could not be fully assessed [38, 39] and may have introduced some bias leading to an over estimation of the seroprevalence. In 79 other studies, authors reported the absence of vaccination in the sampled pigs, while the same information was missing for the remaining 80 studies. However, while vaccines are commonly used in the USA and, to a lesser extent in Europe, their use is not widespread in other parts of the world, often due to the antigenic diversity of the enzootic SIV, the lack of updated knowledge on the circulating strains limiting the design of suitable vaccines and the perceived low economic impact of SI on the swine husbandry [40, 41].

**Fig 5:**
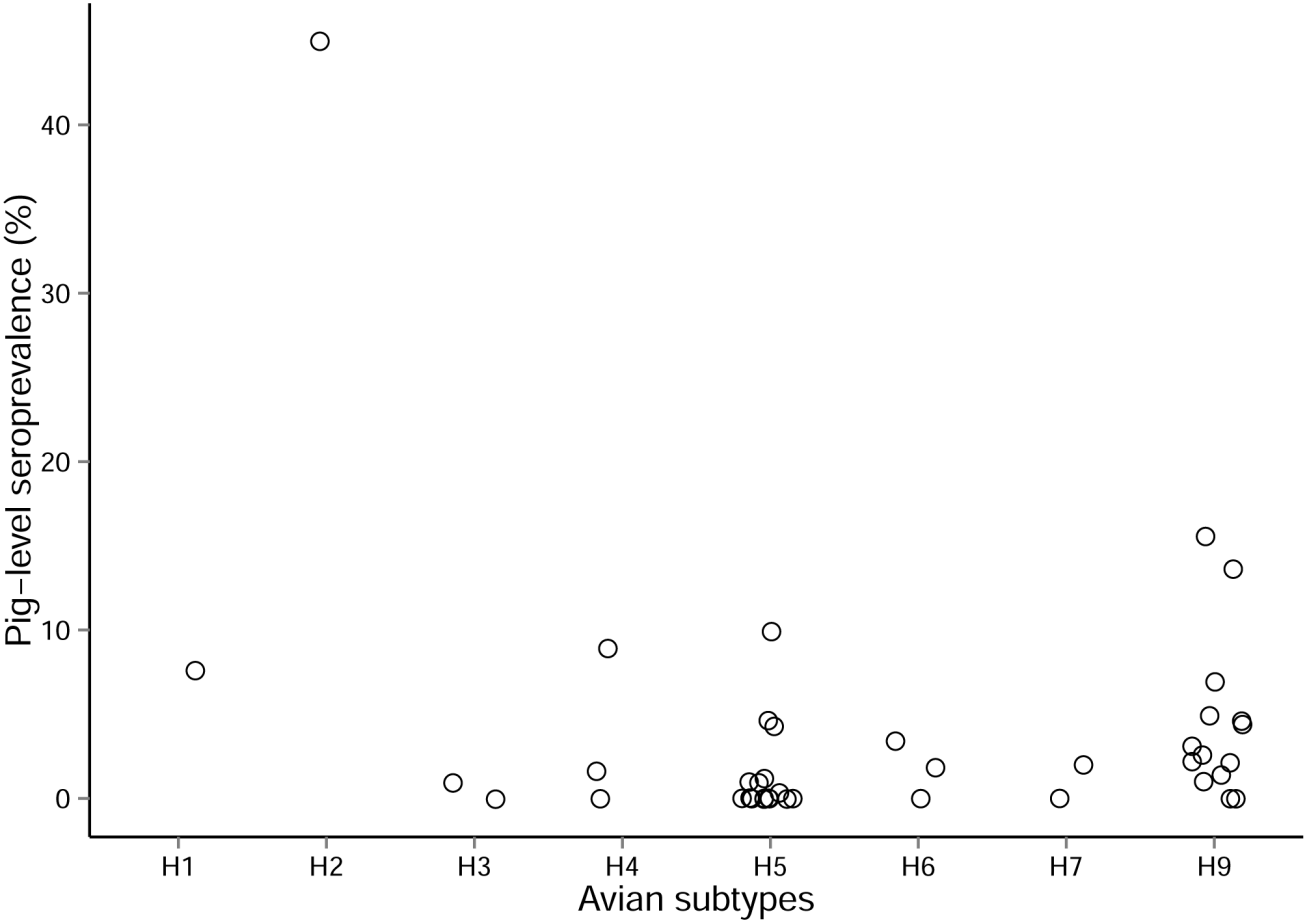
Distribution of the pig-level seroprevalence for avian subtypes. N = 25 studies, 38 entries.

Twenty five studies looked at avian influenza seroprevalence in swine; they were performed mainly in China (n = 16), in other Asian countries (n = 5), the USA (n = 2), and Egypt (n = 2). Subtypes tested were, by order of frequency, H5 and H9 (n>10), then H4, H6, H3, H7, H1 and H2 (n<5) (Fig 5). The seroprevalence values for avian strains were low in general (mean = 4.1%; median = 1.7%) compared to the one for ‘A,H1,H3’ (mean = 32.2%; median = 25.1%). One extreme value was observed for avian strains; an overall prevalence of 45.0% was found in pigs of several categories in two farms for a H2N3 strain in the United States [21]. Avian-like swine H2N3 viruses had previously been isolated from pigs with ILI in these farms, and the high seroprevalence showed that the virus had circulated extensively between pigs in the farms. All the other avian influenza virus seroprevalence values were below 16%. Most of these studies aimed at determining the prevalence of avian viruses in the swine population and suggested sporadic transmission of avian strains rather than persistence in pigs.

#### Meta-regression on pig-level seroprevalence data

Across 39 countries, a total of 137 studies from 83 articles were included in the meta-regression analyses on pig-level seroprevalence data. There were few influenza A or overall H1 and H3 seroprevalence data (‘A,H1+H3’) available for North America and Latin America (S1 Fig). Overall, 79 studies had ‘A,H1+H3’ data, 24 had data on both H1 and H3 subtypes, and 34 had only data for one subtype H1 or H3 (Table 1). There was substantial heterogeneity among the seroprevalence estimates ranging from 0% to 100% and overall means from 32.9% to 54.6%, according to the prevalence value calculated, and with an I^2^ of 99.90% in the random-effects model (value for M1). Influenza A seroprevalence values (‘A,H1+H3’) were relatively high in all continents with means from 32.6% for Africa to 87.8% for Latin America and an overall mean of 49.9% and median of 53.0%; 81.0% of these seroprevalence values were above 20% (S2 Table). In the final mixed-effects models, five variables (type of prevalence data, pig density, continent, GDP, and study size) were significant (p-value<0.05) in at least two models (Table 2). For M1 and M2 the seroprevalence values were significantly lower when only one subtype was tested (‘H1|H3’) compared to the reference group (‘A,H1+H3’) where the seroprevalence was based on testing for influenza A or both H1 and H3. In M1, where only the highest seroprevalence was kept for ‘H1&H3’, the seroprevalence for this group was significantly lower, while in M2, where ‘H1&H3’ was calculated by adding subtype results, there was no significant difference with the ‘H1&H3’ group compared to the reference group. This suggested that adding individual subtype prevalence values (low cross-reactivity between subtypes) was a better approximation compared to taking only the highest value (high cross-reactivity). Also in the three models, seroprevalence was significantly lower for studies with large sample sizes (≥500 pigs). Pig-level seroprevalence data were significantly lower in all models in countries with low (except for M3) or medium pig density compared to those with high pig density. Latin America (M2&M3) and North America (M2) showed significantly higher seroprevalence data compared to Asia, while seroprevalence was lower in Africa (M3). Countries with medium (M3) and low (M1-3) GDP per capita were negatively associated with seroprevalence levels compared to countries with high GDP.

**Table 1:** Number of swine seroprevalence studies and seroprevalence means for the different variables.

Variables	Number of studies (% seroprevalence mean for M1;M2)		
	A,H1+H3	H1&H3	H1|H3
**Pig density**			
High	32 (60.4)	12 (36.8;57.9)	13 (29.6;31.5)
Low	7 (37.4)	4 (40.8;54.6)	9 (13.9;16.9)
Medium	40 (43.8)	8 (27.5;49.6)	12 (50.6;50.6)
**Human density**			
High	26 (46.8)	7 (37.3;65.6)	7 (34.4;34.4)
Low	6 (45.1)	5 (23.1;37.1)	16 (36.3;36.8)
Medium	47 (52.3)	12 (37.4;55.5)	11 (27.0;30.9)
**Continent**			
Asia	20 (38.5)	12 (39.2;60.4)	13 (25.0;26.9)
North America	2 (54.0)	2 (19.6;28.4)	11 (46.7;47.4)
Europe	42 (58.7)	6 (24.7;45.6)	2 (39.3;39.3)
Latin America	2 (87.8)	4 (41.7;63.6)	5 (13.4;13.4)
Africa	13 (32.6)	-	3 (44.5;50.8)
**Gdp per capita**			
High	50 (57.2)	7 (22.8;46.9)	17 (43.1;43.6)
Middle	19 (46.9)	15 (38.7;56.5)	15 (22.6;24.3)
Low	10 (19.3)	2 (42.1;67.0)	2 (22.8;32.1)
**Study size**			
Small	50 (51.9)	11 (45.8;69.4)	17 (40.0;41.1)
Large	29 (46.6)	13 (24.7;42.1)	17 (25.8;27.7)
**Study length**			
Short	71 (51.3)	17 (34.2;52.1)	30 (35.6;37.3)
Long	8 (37.8)	7 (34.9;60.7)	4 (12.7;12.7)
**Period**			
Pre-pdm09	54 (55.0)	16 (31.3;49.7)	18 (40.5;40.9)
Post-pdm09	25 (38.9)	8 (40.5;64.5)	16 (24.3;27.1)
**Premise**			
Slaughterhouse	13 (39.1)	8 (30.0;47.1)	8 (33.9;37.0)
Farm	53 (54.8)	14 (33.4;52.4)	21 (35.2;36.2)
Other/NA	13 (40.8)	2 (58.6;100)	5 (21.3;22.9)
**Overall**	79 (49.9)	24 (34.4;54.6)	34 (32.9;34.4)

A,H1+H3: studies having overall influenza A or aggregated H1 and H3 prevalence data; H1&H3: studies having non-aggregated data on both H1 and H3; H1|H3: studies only having data on one subtype.

Premise category “other/NA” includes entries with missing data, with mixed locations and with seldom mentioned locations such as market or boar testing station.

See S1 Protocol for category description.

**Table 2:** Final mixed-effects models for pig seroprevalence for M1, M2 and M3.

	M1					M2					M3				
	k = 137; R2 = 27.02%					k = 137; R2 = 22.99%					k = 103; R2 = 33.7%				
	Est.	95% CI		p-value		Est.	95% CI		p-value		Est.	95% CI		p-value	
		Lower	Upper				Lower	Upper				Lower	Upper		
Intercept	0.719	0.557	0.880	<.0001	***	0.726	0.552	0.899	<.0001	***	0.805	0.628	0.981	<.0001	***
**A,H1+H3**															
H1&H3	-0.141	-0.262	-0.020	0.023	*	0.064	-0.066	0.193	0.336		-	-	-	-	
H1|H3	-0.201	-0.317	-0.084	0.001	***	-0.176	-0.302	-0.050	0.006	**	-	-	-	-	
**Pig density high**															
Low	-0.225	-0.368	-0.083	0.002	**	-0.181	-0.348	-0.015	0.033	*	-0.079	-0.249	0.092	0.367	
Medium	-0.164	-0.270	-0.057	0.003	**	-0.151	-0.269	-0.032	0.013	*	-0.232	-0.342	-0.122	<.0001	***
**Human density high**															
Low	-	-	-	-		-0.181	-0.425	0.063	0.146		-	-	-	-	
Medium	-	-	-	-		0.014	-0.120	0.148	0.837		-	-	-	-	
**Asia**															
North America	0.186	-0.019	0.392	0.076	.	0.307	0.012	0.603	0.042	*	-0.172	-0.460	0.116	0.242	
Europe	0.012	-0.128	0.152	0.866		-0.009	-0.168	0.150	0.908		-0.024	-0.174	0.126	0.753	
Latin America	0.164	-0.027	0.355	0.093	.	0.265	0.029	0.502	0.028	*	0.411	0.185	0.636	0.000	***
Africa	-0.043	-0.219	0.133	0.631		-0.095	-0.288	0.097	0.332		-0.265	-0.461	-0.069	0.008	**
**Slaughterhouse**															
Farm	-0.014	-0.132	0.104	0.815		-0.011	-0.141	0.119	0.867		-0.031	-0.165	0.103	0.647	
Other/NA	0.068	-0.076	0.212	0.357		0.091	-0.067	0.249	0.258		0.285	0.114	0.457	0.001	**
**Pre-pandemic**															
Post-pandemic	-0.037	-0.144	0.070	0.496		-0.003	-0.120	0.113	0.956		0.012	-0.106	0.130	0.847	
**Gdp high**															
Middle	-0.107	-0.235	0.021	0.100	.	-0.146	-0.324	0.032	0.107		-0.220	-0.361	-0.080	0.002	**
Low	-0.262	-0.435	-0.088	0.003	**	-0.287	-0.480	-0.094	0.004	**	-0.484	-0.681	-0.287	<.0001	***
**Study size small**															
Large	-0.147	-0.235	-0.058	0.001	**	-0.156	-0.251	-0.062	0.001	**	-0.113	-0.215	-0.012	0.028	*

Significance codes:

‘***’ <.001;

0.001≤‘**’<0.01;

0.01≤‘*’<0.05;

0.05≤‘.’<0.1;

‘ ’≥0.1

### Herd-level seroprevalence

#### General description

**Fig 6:**
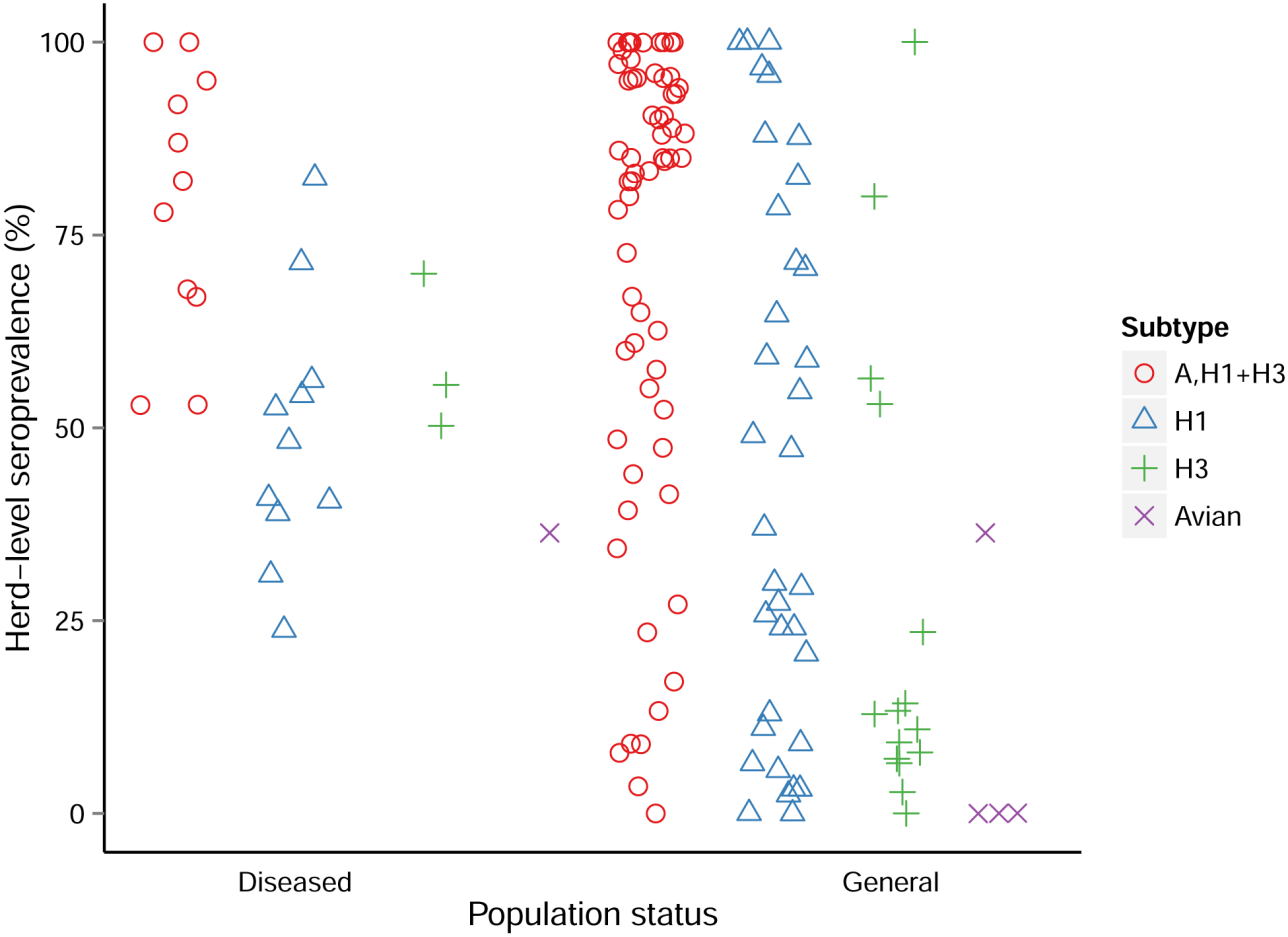
Herd-level seroprevalence distribution by health status and subtypes. N = 51 articles, 112 studies, 143 entries.

In many studies the herd-level seroprevalence was not available. A total of 112 studies with 143 prevalence values were found covering 32 countries in five continents. For the diseased population (20 studies, 26 entries), values ranged from 23.8% to 100% (mean = 62.6%; median = 55.9%), and for the general population values ranged from 0% to 100% with a lower mean = 55.7% but a higher median = 60.0% compared to diseased populations (Fig 6). Five values were available for herd-level seroprevalence for avian influenza viruses and 138 values for ‘A,H1,H3’, i.e. influenza A, swine or human H1 and H3 strains. Overall, prevalence for ‘A,H1,H3’ was usually higher with values from 0% to 100% (mean = 58.5%; median = 61.8%) compared to values for avian strains ranging from 0% to 36.4% (mean = 14.6%; median = 0%). Vaccination was reported in five studies in which sera from vaccinated pigs were excluded [35, 36, 42, 43] or vaccination was limited to 4% of the herds [37]. Serological results from the 2014 article by Panyasing et al. was not included as the sampled herds were vaccinated against SIV [44]. In 61 other studies, vaccination was not used in the farms or the sampled pigs, while in the remaining 46 studies there was no mention of the vaccination status.

The seroprevalence values for avian strains were obtained from studies carried out in China (n = 3), South Korea and Egypt (n = 1 respectively). Three studies showed 0% prevalence for H5N1 (n = 2) and H5 and H9 (n = 1) in farms in China and South Korea. In one of the studies testing for H5N1, H9 was also tested and showed a pig-level seroprevalence of 4.6% (181/3960), however the herd-level seroprevalence was not given for this subtype [45]. Two studies carried out in China and Egypt showed a herd-level seroprevalence of 36.4% (4/11). However, in the study in China, carried out following the isolation of an avian H6N6 virus, the pig-level seroprevalence was only of 3.4% (16/475) [46], and in the study in Egypt, based on a cross-sectional sampling, the pig-level seroprevalence against H5 (H5N1, H5N2) was only of 4.6% (11/240) [47]. These two studies showed that many farms may have pigs with antibodies against avian strains, however in both cases pig-level seroprevalence was very low suggesting sporadic infection rather than persistent circulation between pigs.

#### Meta-regression on herd-level seroprevalence data

For meta-regression analyses, a total of 87 studies from 40 articles were included over 29 countries in five continents (S2 Fig). Overall 61 studies had values for ‘A,H1+H3’, 10 for ‘H1+H3’, and 16 for ‘H1|H3’, with seroprevalence values ranging from 0% to 100% and overall means from 54.9% to 72.8%, according to the prevalence value calculated (Table 3). The heterogeneity was high with an I^2^ of 99.23% (value for M1). Influenza A seroprevalence values (‘A,H1+H3’) were relatively high in all continents with means from 29.3% for Africa to 100% (n = 1) for North America and an overall mean of 72.8% and median of 85%; 88.5% of these seroprevalence values were above 20% (S3 Table). Four variables (pig and human density, continent and GDP) were significantly associated (p-value<0.05) with seroprevalence levels in two or three of the final models (Table 4). Low (M3) and medium (M2) pig densities and a low human density (M1&M2) were associated to lower seroprevalence. Latin America (M1-3) and secondarily North America (M1&M2) had significantly higher seroprevalence compared to Asia, and countries with middle (M1-3) and low (M1-3) GDP showed a negative association.

**Table 3:** Number of the studies and herd seroprevalence means for the different variables.

Variables	Number of studies (% seroprevalence mean for M1;M2)		
	A,H1+H3	H1&H3	H1|H3
**Pig density**			
High	26 (82.9)	3 (55.3;64.3)	5 (80.2;80.2)
Low	6 (31.0)	2 (45.0;74.0)	4 (42.1;55.2)
Medium	29 (72.4)	5 (58.6;76.0)	7 (53.2;54.9)
**Human density**			
High	19 (65.9)	3 (77.8;86.4)	6 (80.5;80.5)
Low	6 (46.1)	4 (62.9;87.0)	8 (43.2;44.2)
Medium	36 (80.9)	3 (21.4;38.0)	2 (56;84.5)
**Continent**			
Asia	5 (52.8)	-	2 (50.4;50.4)
North America	1 (100)	2 (45.0;74.0)	5 (60.1;61.7)
Europe	47 (79)	5 (54.4;63.0)	6 (57.5;57.5)
Latino America	2 (92.3)	2 (80.7;100)	-
Africa	6 (29.3)	1 (25.8;58.1)	3 (64.9;83.8)
**Gdp per capita**			
High	53 (77.2)	5 (64.7;81.4)	13 (61.5;62.1)
Middle	5 (59.7)	5 (45.1;62.8)	1 (88.0;100)
Low	3 (16.6)	-	2 (26.7;49.1)
**Study size**			
Small	35 (80.0)	6 (50.7;66.6)	4 (59.2;70.5)
Large	26 (63.1)	4 (61.2;80.3)	12 (58.7;60.3)
**Study length**			
Short	29 (69.2)	8 (54.6;68.5)	10 (57.1;63.6)
Long	32 (76.0)	2 (56.2;86.8)	6 (61.6;61.6)
**Period**			
Pre-pdm09	47 (82.8)	5 (44.8;60.8)	11 (64.0;64.7)
Post-pdm09	14 (39.2)	5 (65.0;83.5)	5 (47.4;58.7)
**Premise**			
Slaughterhouse	5 (53.4)	1 (59.2;73.5)	2 (56.2;56.2)
Farm	50 (80.2)	8 (58.0;73.7)	12 (57.0;61.4)
Other/NA	6 (26.9)	1 (25.8;58.1)	2 (72.2;78.2)
**Overall**	61 (72.8)	10 (54.9;72.1)	16 (58.8;62.9)

A,H1+H3: studies having overall influenza A or aggregated H1 and H3 prevalence data; H1&H3: studies having non-aggregated data on both H1 and H3; H1|H3: studies only having data on one subtype.

Premise category “other/NA” includes entries with missing data, with mixed locations and with seldom mentioned locations such as market or boar testing station.

See S1 Protocol for category description.

**Table 4:** Final mixed-effects models for herd seroprevalence for M1, M2 and M3.

	M1					M2					M3				
	k = 87; R2 = 58.58%					k = 87; R2 = 49.10%					k = 71; R2 = 60.75%				
	Est.	95% CI		p-value		Est.	95% CI		p-value		Est.	95% CI		p-value	
		Lower	Upper				Lower	Upper				Lower	Upper		
Intercept	0.819	0.501	1.137	<.0001	***	0.717	0.377	1.056	<.0001	***	0.867	0.529	1.205	<.0001	***
**A,H1+H3**															
H1&H3	-0.081	-0.259	0.097	0.371		-	-	-	-		-	-	-	-	
H1|H3	0.116	-0.027	0.258	0.113		-	-	-	-		-	-	-	-	
**Pig density high**															
Low	-0.200	-0.435	0.035	0.095	.	-0.202	-0.447	0.043	0.106		-0.429	-0.682	-0.176	0.001	***
Medium	-0.126	-0.254	0.002	0.054	.	-0.144	-0.281	-0.007	0.039	*	-0.075	-0.186	0.037	0.189	
**Human density high**															
Low	-0.556	-0.816	-0.295	<.0001	***	-0.472	-0.736	-0.208	0.001	***	-	-	-	-	
Medium	0.083	-0.059	0.225	0.254		0.092	-0.057	0.240	0.225		-	-	-	-	
**Asia**															
North America	0.472	0.133	0.810	0.006	**	0.560	0.216	0.903	0.001	**	0.313	-0.032	0.659	0.076	.
Europe	0.024	-0.199	0.248	0.832		0.060	-0.175	0.295	0.618		0.042	-0.177	0.260	0.709	
Latin America	0.950	0.583	1.317	<.0001	***	0.933	0.544	1.322	<.0001	***	0.649	0.310	0.988	0.000	***
Africa	-0.228	-0.528	0.071	0.135		-0.050	-0.360	0.260	0.751		-0.245	-0.548	0.058	0.113	
**Slaughterhouse**															
Farm	0.026	-0.195	0.247	0.818		0.091	-0.144	0.326	0.447		0.012	-0.228	0.253	0.920	
Other/NA	0.130	-0.114	0.373	0.298		0.128	-0.133	0.389	0.337		0.018	-0.272	0.308	0.903	
**Pre-pandemic**															
Post-pandemic	0.075	-0.088	0.238	0.369		0.078	-0.087	0.243	0.352		-0.072	-0.224	0.079	0.351	
**Gdp high**															
Middle	-0.398	-0.608	-0.188	0.000	***	-0.351	-0.556	-0.147	0.001	***	-0.393	-0.568	-0.218	<.0001	***
Low	-0.602	-0.882	-0.323	<.0001	***	-0.512	-0.806	-0.218	0.001	***	-0.377	-0.675	-0.078	0.013	*
**Study size small**															
Large	-0.072	-0.178	0.034	0.185		-0.047	-0.158	0.065	0.412		-0.085	-0.190	0.021	0.115	

Significance codes:

‘***’ <.001;

0.001≤‘**’<0.01;

0.01≤‘*’<0.05;

0.05≤‘.’<0.1;

‘ ’≥0.1

### Virus detection and isolation rate

A total of 170 studies from 133 articles with detection and/or isolation rates were found across 35 countries in six continents. A total of 78 studies reported the detection of H1N1pdm09 and/or their reassortants across 21 countries in the six continents. Outbreak reports of SIV with detection and/or isolation rates were found mainly for Europe (n = 18 studies), and secondly for North America (n = 6), Latin America (n = 5), Asia (n = 4), and finally Oceania (n = 1), while none were found for Africa. Sampling pigs with ILI was reported in the majority of the studies other than outbreaks (N = 136) in North America (77.3%), Europe (72.7%), Oceania (66.7%) and Latin America (58.8%), while most studies did not report targeting pigs with ILI in Africa (72.7%) and Asia (63.9%) (Table 5).

**Table 5:** Number of studies according to continent and ILI status.

	ILI-	ILI+	Total
**Africa**	8 (72.7%)	3 (27.3%)	11
**Asia**	39 (63.9%)	22 (36.1%)	61
**Europe**	6 (27.3%)	16 (72.7%)	22
**Latino America**	7 (41.2%)	10 (58.8%)	17
**North America**	5 (22.7%)	17 (77.3%)	22
**Oceania**	1 (33.3%)	2 (66.7%)	3
**Total**	66	70	136

Outbreaks are excluded. ILI = Influenza-like illness; % of ILI- and + for each continent.

The distributions of the studies according to the detection and isolate rate were left-skewed. Therefore, only univariate analyses were performed on studies other than outbreaks for a few parameters relevant for surveillance, such as sampling in pigs with ILI, premise of sampling, continent and GDP. The random-effects models for detection and isolation rates showed a high heterogeneity (I^2^ = 99.94% and 99.91% respectively). Detection and isolation rates were very high for outbreaks (34 studies) with respective means of 62.6% (22 entries) and 54.9% (20 entries). For other study designs, on average, detection and isolation rates were lower when pigs without symptoms were sampled (means = 4.7% and 4.1% respectively) compared to those in studies only sampling pigs with ILI (means = 20.7% and 10.0% respectively) (Table 6). Both detection and isolation rates in studies sampling pigs with ILI were significantly higher (estimates = 0.148, 0.054; p = 0.0007, 0.002 respectively) compared to rates in studies sampling apparently healthy pigs; sampling both ILI+/- pigs was only significant for detection rates (est. = 0.100; p = 0.033). Studies performed in Europe showed positive associations for both detection and isolation rates (est. = 0.123, 0.090; p = 0.05, 0.0004) and in North America for isolation rate only (est. = 0.109; p<0.0001). Similarly, low GDP was negatively associated with both rates (est. = -0.135, -0.079; p = 0.04, 0.005) and middle GPD with isolation rate only (est. = -0.047; p = 0.005). As shown previously, in continents with generally higher GDP per capita such as Europe, North America and secondarily Latin America, studies were mainly focusing on sampling pigs with ILI compared to Asia and Africa. The premise variable was not significantly associated with detection or isolation rates (p>0.05).

**Table 6:** Number of studies and means of detection and isolation rates for different variables (outbreaks are excluded).

Variables	Number of studies (% mean)	
	Detection rate	Isolation rate
**ILI**		
No	24 (4.7)	53 (4.1)
Yes	25 (20.7)	34 (10.0)
Both ILI and Non-ILI pigs sampled	19 (15.5)	8 (5.3)
**Premise**		
slaughterhouse	7 (13.1)	26 (3.8)
farm	51 (13.3)	51 (7.0)
other/NA	10 (15.4)	18 (8.1)
**Continent**		
Asia	12 (7.9)	58 (3.1)
North America	18 (12.2)	10 (14.8)
Europe	14 (20.7)	10 (12.3)
Oceania	3 (20.7)	-
Latino America	13 (18.3)	9 (7.9)
Africa	8 (2.6)	8 (9.2)
**Gdp per capita**		
high	39 (14.8)	37 (9.4)
middle	22 (15.7)	49 (5.0)
low	7 (0.5)	9 (0.8)
**Overall**	68 (13.6)	95 (6.3)

### Risk factors

A total of 20 articles were retrieved for studies with risk factor analysis carried out on or after 1990 in 13 countries in Africa, Asia, Europe and North America. Most studies estimated prevalence of influenza based on serological results (n = 14), but some relied on virological results (n = 6). Studies were carried out mainly in farms (n = 18), but also in both farms and slaughterhouses (n = 1) or in agricultural fairs (n = 1). Factors related to the swine population, e.g. pig density in an area or number of pigs in a barn, were found significant in almost two third of the articles (Table 7). Other factors related to farm management, biosecurity and housing were also consistently associated with influenza A infection.

**Table 7:** Categories of variables significantly associated with influenza circulation.

Category	Number of articles	Number of variables
Swine population	13	26
Farm management	9	14
Biosecurity	5	14
Housing	4	5
Human contact	3	3
Environmental factors	3	6
Other animal species	2	7
Clinical observations	2	3
Geography	2	2
Other	1	1
**Total**	20*	**81**

* Total number of articles; several variables were treated per article.

Higher herd and pig densities and higher number of pigs in farms or agricultural fairs were associated with higher influenza prevalence in 10 articles (S1 Table, Ref 16, 50, 58, 107, 108, 112, 118, 148, 149, 182 in S1 References). Only one article in Cambodia showed a negative association between pig density and influenza prevalence (S1 Table, Ref 124 in S1 References); authors suggested this may be due to a higher number of commercial farms with better biosecurity in areas with high pig density or to spatial bias resulting from the non-representative sample. Two articles showed differences in influenza prevalence according to certain categories of pigs, such as higher prevalence in piglets and newly introduced gilts compared to onsite gilts, suggesting infection was more likely to occur at certain production stages (young and potentially naïve animals in this case) in large commercial farms in the USA (S1 Table, Ref 46, 76 in S1 References).

Within farm management risk factors, the purchase of pigs was systematically associated with higher influenza prevalences (S1 Table, Ref 107, 182 in S1 References). Factors related to sow management such as the use of an external source of gilts (univariate), the sow replacement rate and the parity of sows also showed positive association (S1 Table, Ref 149, 172 in S1 References). In three studies, finisher farms had lower odds to be infected (S1 Table, Ref 41, 149, 186 in S1 References), and in another study the transfer of young fattening pigs through a room with older pigs increased the odds of infection (S1 Table, Ref 50 in S1 References). This showed once again the importance of influenza transmission between different age groups within farms and the impact of farm management on disease circulation. Other factors were described in the farm management category but the observations were based on univariate analysis.

Biosecurity measures were reported in five articles (S1 Table, Ref 50, 65, 114, 172, 182 in S1 References), usually showing higher influenza prevalence associated with low biosecurity such as the lack of all-in all-out practices or limited duration of the empty period between batches in certain groups of pigs, uncontrolled access to the farm, and lack of bird-proofs nets. In Malaysia, the odds of disease increased seven times when carcasses of dead pigs were handled by the authorities compared to being buried by the farmers, probably due to a more important movement of personal in and out of the farm (S1 Table, Ref 182 in S1 References). In univariate analysis, vaccination against SIV but also PRRS and PCV2 seemed beneficial, while separating diseased pigs in special units showed contradictory results.

Housing factors were found in four articles. Factors increasing the density (e.g. low floor space per pig) and the contact between pigs (e.g. discontinuous partition between pens, pigs kept indoor) inside the farms were positively associated with influenza prevalence (S1 Table, Ref 50, 112, 172 in S1 References). Also the type of floor (e.g. slatted vs straw) seemed to have an impact on disease circulation (S1 Table, Ref 107, 112 in S1 References). Similarly, some environmental factors inside the farms such as temperature and temperature control showed associations with influenza prevalence, suggesting that the control of environmental parameters in farms may be important (S1 Table, Ref 50 in S1 References). Weather parameters were also mentioned, with higher seroprevalence associated with higher temperature and higher wind speed in the USA, and sampling outside the summer season in the UK (S1 Table, Ref 41, 112 in S1 References).

Risk factors linked to human health, activity and density were also investigated. For H1N1pdm09, a study showed the presence of ILI in the farm staff increased the odds of influenza in pigs in Norway by fourfold (S1 Table, Ref 58 in S1 References). In Cambodia, human density was positively associated with H3N2 prevalence in swine (S1 Table, Ref 124 in S1 References). On the contrary, in Vietnam, the employment of external swine workers showed a negative association with SI; the authors did not describe sufficient information to offer a likely explanation for this result (S1 Table, Ref 186 in S1 References), e.g. whether farms where external workers were employed had better biosecurity practices. Factors related to the presence of other species such a birds or pets were also examined. In Cameroon, researchers used random forest analyses and showed that the three best predictors for the presence of H1N1pdm09 in swine were different contact rates between free-ranging swine, domestic and wild birds, and humans (S1 Table, Ref 93 in S1 References). In Malaysia, the authors showed that the presence of mammalian pets such as cats was positively associated with influenza, while the presence of birds had a negative association (S1 Table, Ref 182 in S1 References).

The presence of certain disease symptoms in herds such as ILI (univariate) and of breeding shows in agricultural fairs increased the odds of influenza infection (S1 Table, Ref 16, 114, 121 in S1 References). A regional effect was observed in the USA, and also in Bhutan with East and East-Central regions being negatively associated with disease prevalence, and explained by the authors by the remoteness of the eastern regions (S1 Table, Ref 76, 118 in S1 References).

## Discussion

The objectives of this systematic review were to investigate the epidemiological characteristics of SI across different countries and to highlight factors that were important for SIV isolation and therefore for SI surveillance. This systematic review included all articles found in scientific public databases which reported data for SI such as pig-level and herd-level seroprevalence, isolation and detection rates, and risk factors in natural settings and from studies carried out on or after 1990. A total of 884 abstracts and 623 full-text articles on swine influenza were screened, and 217 articles were finally included in the analysis. Only 49 articles were excluded because they were not retrieved or because of the language, with 10 of them probably being reviews. Only two articles about SIV from Oceania were retrieved but one was excluded because of the lack of relevant data for this systematic review; before the 2009 pandemic countries such as Australia were free of swine influenza [8, 48]. A limitation of the search was that grey literature, such as reports from national and international institutions or surveillance networks, was not included.

Study design and reporting were rather heterogeneous and this may have introduced some biases in the analyses. The measure and reporting of seroprevalence with results given for either influenza A, specific subtypes or strains which could not always be aggregated; several models with different assumptions were therefore built for the meta-regression analyses. Also the time frames were different across the studies (from a few months to several years). For some virological studies, it was not always clear whether the isolation rate reported was an overall rate or a partial rate (i.e. only for a certain subtype). Often study designs were not very clear, and most studies were not representative; sampling was often not randomized and convenience sampling was performed. Many data on study variables such as farm category (e.g. familial, industrial, small or large), production systems (e.g. breeding, finishing, mixed), pig category or age and local pig density (e.g. regional or county level) were missing and could not be included in meta-regression analyses. However, such variables were commonly studied in articles focusing on risk factors. As a result, country-level variables were used in the statistical models; however data such as the pig density in a country are not very precise because of heterogeneity in the pig distribution. The vaccination status was not always described in serological studies; it could be assumed that vaccination was non-existent or rare when authors did not mention it, as it would constitute an obvious bias and SIV vaccination is very limited worldwide except in the USA and some European countries [40]. In general, a more clear and detailed description of study design, results and limitations are recommended for future studies. Guidelines for reporting seroepidemiological studies have been described for human influenza and could be developed for swine influenza epidemiological and surveillance studies [49].

The results of the systematic review showed that many studies were performed in Asia and secondly in Europe, encompassing data from a large number of countries; many articles (>10) were found for China, the USA, South Korea, and Brazil. Seroprevalence data were available for avian strains mainly in Asia. Most studies reported low seroprevalence values for the different avian strains tested suggesting a limited transmission of influenza viruses between avian and swine and within swine herds. Two studies reported a relatively high avian influenza herd seroprevalence (36.4%) on a limited number of herds (N = 11) for H6N6 in China and H5 in Egypt, although pig seroprevalence values were low [46, 47]. This suggested that spillovers from avian to swine may be frequent in some places but that the avian viruses do not transmit extensively between pigs. Some exceptions were reported, for example with one study finding a very high pig seroprevalence for avian influenza H2N3 (45.0%) in the USA, showing the potential for some avian strains to transmit efficiently in pigs [21]. Infections of H5N1 and H7N9 AIV in humans have been frequently detected in Asia causing severe illness and deaths; however, so far there have been no or very limited human-to-human transmission, and the majority of the cases were tied to poultry exposure [50, 51]. Similarly to pigs, most studies have reported null or low AIV seroprevalence in the human population even in high risk situations (AIV occurrence in poultry, poultry exposed persons…). Studies from China reported low human seroprevalence for H7N9 with values of 0% [52, 53] and >6% in poultry workers [54]. A 2011 systematic review reported mainly low H5N1 seroprevalence in humans from 0–3.1% in several countries in Asia and in Nigeria and Germany; two higher seroprevalence of 10 and 12% were found in Hong Kong in 1997 in poultry workers and household contacts of H5N1 cases respectively [55]. In Egypt, values of 8.7% (HI test) and 14% (ELISA test) were reported from humans in hotspots [56]. These values were very similar compared to the pig-level seroprevalence found for H7 (0–2%) and H5 (0–9.9%) (Fig 5). H9N2 seroprevalence in humans with poultry exposure had a median of 9% (1–43%) by HI test and 2.7% (0.6–9%) by MN test according to another systematic review encompassing results from Asia, the Middle East, North America, Africa and Europe [57]. Pig-level seroprevalence for H9 (0–15.6%) were usually higher compared to H5 and H7.

Regarding subtypes circulating commonly in pigs, i.e.H1N1, H1N2, and H3N2, a high variability in the seroprevalence values was noted in the general population, but high values were observed in all continents with pig and herd-level seroprevalence means of 49.9% and 72.8% respectively for the overall influenza values (‘A,H1+H3’). Lower pig and herd-level seroprevalence means were found for Africa (32.6%; 29.3%) and secondarily Asia (38.5%; 52.8%) compared to other continents (Tables 1 and 3). Some study variables such as farm category, production type, biosecurity and pig subpopulation could not be included in meta-regression analyses as there were too many missing data. However, some of these variables were analyzed in risk factor articles. The meta-regression analyses using pig and herd-level seroprevalence showed consistent results regarding the association of country related variables with seroprevalence. Seroprevalence values were significantly lower in countries with low and medium pig density, low and middle GDP, and secondly low human density (herd-level seroprevalence only). The association between high pig densities (in terms of number of pig herds and number of pigs in an area) and high SI prevalence was also shown in risk factor articles from several continents (S1 Table). Indeed, a dense swine population is suitable to the spread of infectious diseases such as influenza which can be transmitted directly from pig to pig, by fomites and probably also by aerosols from farm to farm [58, 59]. For example, in Vietnam no SIV was detected serologically in Northern provinces where the pig density is very low [60], while higher seroprevalence levels were detected in areas near Hanoi, an area of very high pig density [61, 62]. A higher number of pigs per farm was also a factor commonly associated with higher influenza prevalence in articles from North America, Europe and Asia. This association was also found in a more recent study carried out in Vietnam showing higher isolation rates in large corporate farms with more than 1000 pigs compared to smaller corporate and family-operated farms [63]. One hypothesis to explain the negative association with the GDP per capita is that in countries with higher GDP, swine are more commonly raised in larger industrial farms, which have larger numbers of pigs and a higher within-farm pig density, compared to countries with lower GDP where familial farming may be more common. Also, countries with lower GDP might have SIV detection issues due to less advanced and less suited laboratory techniques or antigens not matching the local strains. The SI seroprevalence were also significantly higher in Latin America and secondarily in North-America compared to Asia, where industrial farming is the most common method while in Asia familial farming is still important [64]. This also suggests that despite better biosecurity levels often being found in industrial farming, influenza is difficult to control in large swine herds. Nevertheless, the risk factor studies showed that some biosecurity practices such as all-in all-out practices were negatively associated with SIV prevalence; factors such as housing and temperature control were also shown to have an impact, together with pig trades. Risk factor articles showed associations between prevalence and respectively the production type and the pig subpopulation. Finisher only farms showed lower SIV prevalence in studies in Vietnam [61] and North America [65, 66]. Several studies showed higher prevalence in young pigs (piglets [67, 68] and neonatal pigs [69] in the USA, 3 weeks to 4.5 months old in large farms in Vietnam [63]); other studies mentioned detecting systematic infection in the nursery at around 50 days of age in two farms in France [70], and SIV shedding in piglets at 3–4 weeks of age in a farm in Spain [71]. These observations suggest an enzootic circulation of SIV in many pig farms specialized in breeding, breed-to-wean, farrow-to-finish or wean-to-finish where young pigs are constantly introduced (especially in large farms) and may allow the maintenance of SIV circulation.

Low and middle GDP were also negatively associated with isolation and detection rates, while Europe and secondarily North America showed positive associations. Limits in sampling techniques, sample handling in the field and laboratory capacities could also explain lower virus isolation. Sampling pigs with ILI during routine surveillance or outbreaks was positively associated with virus detection and isolation. Clinical surveillance of SIV in ILI pigs could be effective despite the high number of respiratory diseases causing ILI in swine (e.g. PRRS). Countries with more robust surveillance systems allowing early detection of ILI clinical signs and with better laboratory capacities may have an advantage for SIV isolation. Targeting pigs with ILI could be more efficient although it does not allow the detection of some influenza viruses circulating asymptomatically and of potential pandemic importance, as shown with the example of low pathogenic and asymptomatic H7N9 in poultry [72]. Pandemic risk is not necessarily limited to viruses that cause symptoms in the reservoir animal species. Thus, SI surveillance of asymptomatic animals is probably also important for obtaining a comprehensive understanding of SIV of pandemic risk. Moreover, as mentioned previously, SIV circulation differs according to the farming system, production type and pig subpopulation, and such information is valuable to target sampling in the pig population. Based on the results of this review, targeting young pigs such as weaners in large farms seems the most appropriate way to increase the probability of virus isolation. However, farming practices may differ across countries; such risk factors should be re-evaluated for each situation.

This comprehensive review has highlighted the importance of key risk factors such as pig density and intensive breeding systems in the circulation of SIV. Additional studies would be required to identify relevant prevention and control measures that can be implemented, especially in settings which already have high biosecurity levels to prevent the spread of SIV. This work also highlighted the limits of current surveillance systems and surveillance data quality available to conclude on SIV circulation patterns in many low-to-middle GDP countries. In countries with limited resources, as a minimum, surveillance systems could be developed by targeting swine with ILI and by setting up risk-based surveillance systems targeting specific swine age groups and farming systems to improve SIV detection sensitivity at reasonable costs.

## Supporting information

S1 Checklist PRISMA 2009 checklist. (DOC)

S1 Fig Pig-level seroprevalence data according to the continent and subtype. N = 83 articles, 137 studies, 205 entries.
(PDF)

S2 Fig Herd-level seroprevalence data according to the continent and subtype. N = 40 articles, 89 studies, 113 entries (two studies and entries later excluded as no sample size data).
(PDF)

S1 Protocol Details on material and methods. (DOCX)

S1 References Full list of the 205 references included in the systematic review. (DOCX)

S1 Table Risk factors associated with influenza circulation in studies carried out on or after 1990. (DOCX)

S2 Table Pig seroprevalence studies database. (XLSX)

S3 Table Herd seroprevalence studies database. (XLSX)

S4 Table Isolation and detection rates studies database. (XLSX)
