# Supplementary figures and images for: Epidemiological features of influenza circulation in swine populations: A systematic review and meta-analysis
Source: PLoS One. 2017 Jun 7;12(6):e0179044. doi: 10.1371/journal.pone.0179044 (PMC5462427; doi:10.1371/journal.pone.0179044)

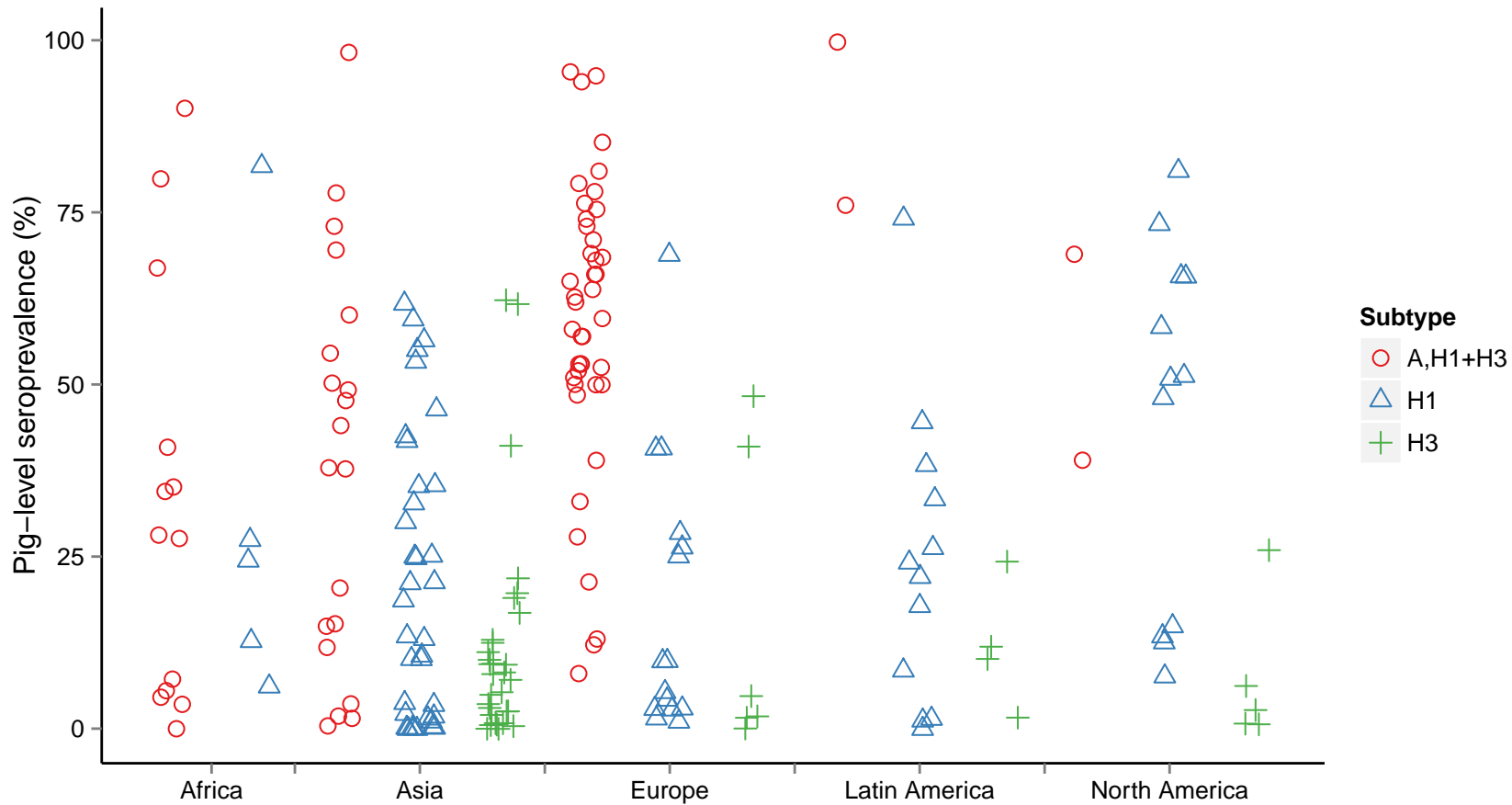

Supplement: S1 Fig — N = 83 articles, 137 studies, 205 entries. (PDF) [file pone.0179044.s002.pdf]

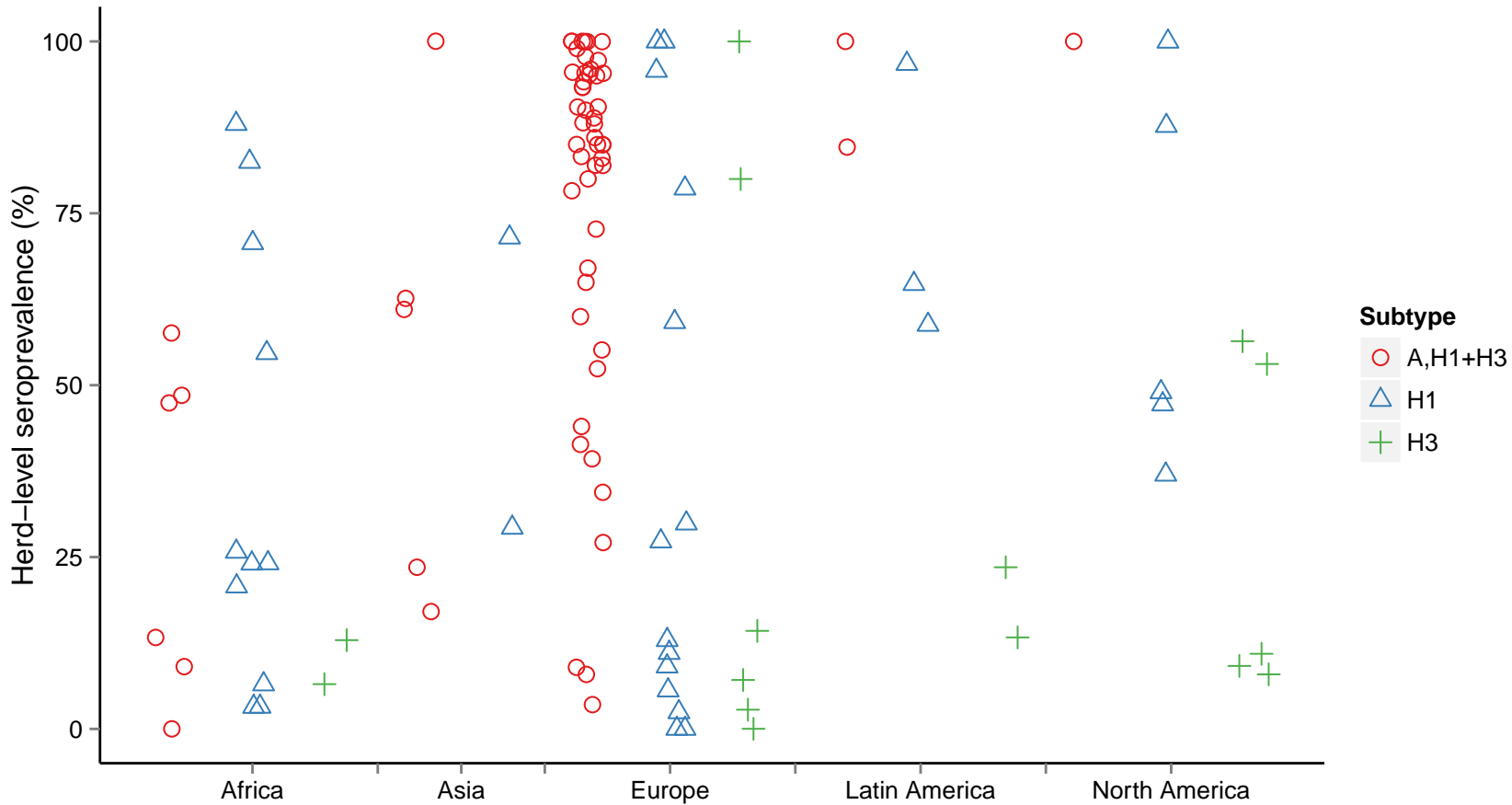

Supplement: S2 Fig — N = 40 articles, 89 studies, 113 entries (two studies and entries later excluded as no sample size data). (PDF) [file pone.0179044.s003.pdf]
