# Supplementary material for: Epidemiological features of influenza circulation in swine populations: A systematic review and meta-analysis
Source: PLoS One. 2017 Jun 7;12(6):e0179044. doi: 10.1371/journal.pone.0179044 (PMC5462427; doi:10.1371/journal.pone.0179044)
Supplement: S1 Protocol — (DOCX) [file pone.0179044.s004.docx]

**Epidemiological features of influenza circulation in swine populations: a systematic review and meta-analysis**

Eugénie Baudon, Marisa Peyre, Malik Peiris, Benjamin J. Cowling

**S1 Protocol. Details on material and methods**

**Search strategy**

The search terms were divided into two main parts (see table below). The first part was related to the host population, i.e. swine, and words associated to swine, mammals and animals in general were included; for this purpose synonyms were hand searched. The search of these terms was limited to the title of the articles. The second part was related to the disease; two general terms "influenza" and "flu" were chosen and looked for in all fields of the articles, except in Scopus and Web of Science where only titles, abstracts and keywords were searched. In PubMed and the other databases when possible, an additional search based on MeSH and keywords respectively was done using the general terms "swine" and "influenza" to make sure no articles with these two keywords were missed. All the references were imported into EndNote® X7 (Thomson Reuters, New York, NY, USA).

Search terms and number of references for each online database

| **PubMed** | **3,322 references** |
| --- | --- |
| (((swine[Title] OR pig*[Title] OR piglet*[Title] OR gilt*[Title] OR boar*[Title] OR sow*[Title] OR weaner*[Title] OR hog*[Title] OR porcine[Title] OR pork*[Title] OR "Sus scrofa"[Title] OR "Sus domesticus"[Title] OR "suidae"[Title] OR livestock[Title] OR herd*[Title] OR farm*[Title] OR flock*[Title] OR mammal*[Title] OR animal*[Title] OR zoo*[Title]) NOT ("guinea pig"[Title] OR "guinea pigs"[Title])) AND (influenza OR flu)) OR (("Swine*"[MeSH]) AND ("Influenza A virus*"[MeSH]))  [AND ("2014/10/13"[Date - Publication] : "3000"[Date - Publication])] | |
| **Web of Science** | **3,652 references** |
| TITLE:(( swine OR pig* OR piglet* OR gilt* OR boar* OR sow* OR weaner* OR hog* OR porcine OR pork* OR "Sus scrofa" OR "Sus domesticus" OR "suidae" OR livestock OR herd* OR farm* OR flock* OR mammal* OR animal* OR zoo*) NOT ("guinea pig" OR "guinea pigs"))  AND TOPIC:(influenza OR flu)  (note: no "All fields" nor "keywords" only, "topic" relates to title, abstract and keywords)  [Year: from 2014 to 2016] | |
| **ScienceDirect** | **4,581 references** |
| The two searches below were done separately and then imported with exclusion of duplicates. (TITLE((swine OR pig* OR piglet* OR gilt* OR boar* OR sow* OR weaner* OR hog* OR porcine OR pork* OR "Sus scrofa" OR "Sus domesticus" OR "suidae" OR livestock OR herd* OR farm* OR flock* OR mammal* OR animal* OR zoo*) AND NOT ("guinea pig" or "guinea pigs")) AND (flu OR influenza)) AND (KEYWORDS(swine*) and KEYWORDS(influenza*))  [Year: from 2014 to Present] | |
| **Scopus** | **5,492 references** |
| ((TITLE(swine OR pig* OR piglet* OR gilt* OR boar* OR sow* OR weaner* OR hog* OR porcine OR pork* OR "Sus scrofa" OR "Sus domesticus" OR "suidae" OR livestock OR herd* OR farm* OR flock* OR mammal* OR animal* OR zoo*) AND NOT TITLE("guinea pig" OR "guinea pigs")) AND TITLE-ABS-KEY (flu OR influenza)) OR (KEY(swine*) AND KEY( influenza*))  [Filter: 2014, 2015, 2016] | |

[Additional terms or selection for the search update on 18 January 2016].

**Study selection**

For seroprevalence data, articles sampling only one farm were excluded (e.g. articles on outbreaks with limited sample size). For isolation and detection rates, articles reporting outbreaks were included when at least two pigs were sampled. The study design was classified as an outbreak investigation when diseased pigs were sampled promptly in farms where a recent increase in the number of ILI cases was observed. Articles with isolation/detection rate only for specific subtypes were excluded; however, studies were kept when the subtype or strain were clearly the only one circulating, e.g. for an outbreak with identification of the subtype, or in countries where only a certain strain had been detected in swine populations (e.g. H1N1pdm09 in Norway). When the same results were published in several articles, only those with the most complete data and/or the most recently published were kept. Reviews were all screened as full texts but were excluded in the last step as they did not contain original data.

**Data extraction and analysis**

In serological studies, when sera were tested for both H1 and H3 using representative antigens for the location, the overall seroprevalence for influenza A was recorded when possible, i.e. when the number of sera positive for H1, H3 and for both H1 and H3 was given. In some virological studies, only the positive RT-PCR samples were tested by isolation, since the RT-PCR was believed to be more sensitive. In these cases, the overall isolation rate taking into account the original sample size (RT-PCR positive and negative samples) was recorded.

Regarding detection or isolation rates, sometimes several entries were recorded for a same study, e.g. case control study, when a group of pigs was sick and the other apparently healthy, in order to test the association between influenza rates and presence of ILI or of lung lesions in the sampled pigs. In retrospective studies using samples that had been submitted to diagnostic laboratories, the pigs were considered as sick. Some studies used lesions in lungs regardless of observed symptoms; these were classified in the category ‘with ILI’. When the health status was not described, it was considered that the pigs were probably in majority apparently healthy and it was analyzed as such.

For the meta-regression analyses, the study variables were selected based on their relevance and on sufficient data availability. There were many missing data for most study variables such as the size of premises, the type of production, the category or age of pigs sampled, and biosecurity levels; as a result, such variables were not included in the meta-regression analyses. A total of five study variables were included: ‘disease test’ with three categories according to the seroprevalence values available (‘A,H1+H3’, ‘H1&H3’, and ‘H1|H3’), ‘premise’ according to the location of sampling, ‘period’ depending on whether the study was performed before or after the 2009 pandemic, ‘study size’ depending on whether the number of pigs or herds sampled was small (<500 pigs or <40 herds) or large (≥500pigs or ≥40 herds) based on the median, and ‘study length’ depending on whether the length of the study was short (≤2 years for pig-level and <2 years for herd-level) or long (>2 years for pig-level or ≥2 years for herd-level) also based on the median. For country variables, pig and human densities were calculated for each country. A total of four country variables were included. Categorical variables were defined for pig and human densities and GDP per capita. For the first two variables, the 1st and 3rd quantiles were used to separate the variables into three categories; for this purpose the data from the countries for all the studies included in this review were used. For the GDP, the limits used were ‘<2,000USD/ capita/year’ and ‘≥20,000USD/capita/year’. The continent where the study was located was also included. Since only studies in South and East Asia were found (none in the Central Asia/Middle East), Israel was included in the “Africa” continent for geographical reasons. Also, the French Island Reunion was considered as a separate entity and located in Africa.
