# Supplementary material for: Epidemiological features of influenza circulation in swine populations: A systematic review and meta-analysis
Source: PLoS One. 2017 Jun 7;12(6):e0179044. doi: 10.1371/journal.pone.0179044 (PMC5462427; doi:10.1371/journal.pone.0179044)
Supplement: S1 References — (DOCX) [file pone.0179044.s005.docx]

**Epidemiological features of influenza circulation in swine populations: a systematic review and meta-analysis**

Eugénie Baudon, Marisa Peyre, Malik Peiris, Benjamin J. Cowling

**S1 References. Full list of the 217 references included in the systematic review**

These reference numbers were used in S1 Table, but not in the article.

1. Abe H, Mine J, Parchariyanon S, Takemae N, Boonpornprasert P, Ubonyaem N, et al. Co-infection of influenza A viruses of swine contributes to effective shuffling of gene segments in a naturally reared pig. Virology. 2015;484:203-12.

2. Adeola OA, Adeniji JA, Olugasa BO. Isolation of influenza A viruses from pigs in Ibadan, Nigeria. Vet Ital. 2009;45(3):383-90.

3. Adeola OA, Adeniji JA, Olugasa BO. Detection of haemagglutination-inhibiting antibodies against human H1 and H3 strains of influenza A viruses in pigs in Ibadan, Nigeria. Zoonoses Public Health. 2010;57(7-8):e89-94.

4. Allerson MW, Davies PR, Gramer MR, Torremorell M. Infection Dynamics of Pandemic 2009 H1N1 Influenza Virus in a Two-Site Swine Herd. Transbound Emerg Dis. 2013.

5. Amorim AR, Fornells LA, Reis Fda C, Rezende DJ, Mendes Gda S, Couceiro JN, et al. Influenza A virus infection of healthy piglets in an abattoir in Brazil: animal-human interface and risk for interspecies transmission. Mem Inst Oswaldo Cruz. 2013;108(5):548-53.

6. Andreasen M, Nielsen JP, Baekbo P, Willeberg P, Botner A. A longitudinal study of serological patterns of respiratory infections in nine infected Danish swine herds. Prev Vet Med. 2000;45(3-4):221-35.

7. Awosanya EJ, Ogundipe G, Babalobi O, Omilabu S. Prevalence and correlates of influenza-A in piggery workers and pigs in two communities in Lagos, Nigeria. The Pan African medical journal. 2013;16:102.

8. Barigazzi G, Donatelli I. Swine influenza in Italy. Veterinary research communications. 2003;27 Suppl 1:93-9.

9. Baudon E, Poon LL, Dao TD, Pham NT, Cowling BJ, Peyre M, et al. Detection of Novel Reassortant Influenza A (H3N2) and H1N1 2009 Pandemic Viruses in Swine in Hanoi, Vietnam. Zoonoses Public Health. 2015;62(6):429-34.

10. Beaudoin A, Johnson S, Davies P, Bender J, Gramer M. Characterization of influenza a outbreaks in Minnesota swine herds and measures taken to reduce the risk of zoonotic transmission. Zoonoses Public Health. 2012;59(2):96-106.

11. Bi Y, Fu G, Chen J, Peng J, Sun Y, Wang J, et al. Novel swine influenza virus reassortants in pigs, China. Emerg Infect Dis. 2010;16(7):1162-4.

12. Bikour MH, Frost EH, Deslandes S, Talbot B, Elazhary Y. Persistence of a 1930 swine influenza A (H1N1) virus in Quebec. J Gen Virol. 1995;76 ( Pt 10):2539-47.

13. Bikour MH, Frost EH, Deslandes S, Talbot B, Weber JM, Elazhary Y. Recent H3N2 swine influenza virus with haemagglutinin and nucleoprotein genes similar to 1975 human strains. J Gen Virol. 1995;76 ( Pt 3):697-703.

14. Bliss N, Nelson SW, Nolting JM, Bowman AS. Prevalence of Influenza A Virus in Exhibition Swine during Arrival at Agricultural Fairs. Zoonoses Public Health. 2016.

15. Bowman AS, Nolting JM, Nelson SW, Slemons RD. Subclinical influenza virus A infections in pigs exhibited at agricultural fairs, Ohio, USA, 2009-2011. Emerg Infect Dis. 2012;18(12):1945-50.

16. Bowman AS, Workman JD, Nolting JM, Nelson SW, Slemons RD. Exploration of risk factors contributing to the presence of influenza A virus in swine at agricultural fairs. Emerging Microbes and Infections. 2014;3.

17. Bowman AS, Nelson SW, Page SL, Nolting JM, Killian ML, Sreevatsan S, et al. Swine-to-Human Transmission of Influenza A(H3N2) Virus at Agricultural Fairs, Ohio, USA, 2012. Emerg Infect Dis. 2014;20(9):1472-80.

18. Brown IH, Alexander DJ, Chakraverty P, Harris PA, Manvell RJ. Isolation of an influenza A virus of unusual subtype (H1N7) from pigs in England, and the subsequent experimental transmission from pig to pig. Vet Microbiol. 1994;39(1-2):125-34.

19. Brown IH, Chakraverty P, Harris PA, Alexander DJ. Disease outbreaks in pigs in Great Britain due to an influenza A virus of H1N2 subtype. Vet Rec. 1995;136(13):328-9.

20. Brown IH, Harris PA, Alexander DJ. Serological studies of influenza viruses in pigs in Great Britain 1991-2. Epidemiol Infect. 1995;114(3):511-20.

21. Campitelli L, Donatelli I, Foni E, Castrucci MR, Fabiani C, Kawaoka Y, et al. Continued evolution of H1N1 and H3N2 influenza viruses in pigs in Italy. Virology. 1997;232(2):310-8.

22. Cao N, Zhu W, Chen Y, Tan L, Zhou P, Cao Z, et al. Avian influenza A (H5N1) virus antibodies in pigs and residents of swine farms, southern China. J Clin Virol. 2013;58(4):647-51.

23. Cardinale E, Pascalis H, Temmam S, Herve S, Saulnier A, Turpin M, et al. Influenza A(H1N1)pdm09 Virus in Pigs, Reunion Island. Emerg Infect Dis. 2012;18(10):1665-8.

24. Carman S, Stansfield C, Weber J, Bildfell R, Van Dreumel T. H3N2 influenza A virus recovered from a neonatal pig in Ontario--1997. The Canadian veterinary journal La revue veterinaire canadienne. 1999;40(12):889-90.

25. Charoenvisal N, Keawcharoen J, Sreta D, Chaiyawong S, Nonthabenjawan N, Tantawet S, et al. Genetic characterization of Thai swine influenza viruses after the introduction of pandemic H1N1 2009. Virus Genes. 2013;47(1):75-85.

26. Chatterjee S, Mukherjee KK, Mondal MC, Chakravarti SK, Chakraborty MS. A serological survey of influenza a antibody in human and pig sera in Calcutta. Folia microbiologica. 1995;40(3):345-8.

27. Chen Y, Zhang J, Qiao C, Yang H, Zhang Y, Xin X, et al. Co-circulation of pandemic 2009 H1N1, classical swine H1N1 and avian-like swine H1N1 influenza viruses in pigs in China. Infect Genet Evol. 2013;13:331-8.

28. Chen J, Fu X, Chen Y, He S, Zheng Y, Cao Z, et al. Identification of four genotypes of H3N2 swine influenza virus in pigs from southern China. Arch Virol. 2014;159(10):2705-9.

29. Chiapponi C, Baioni L, Luppi A, Moreno A, Castellan A, Foni E. Temporal insight into the natural generation of a new reassortant porcine influenza virus in a swine holding. Vet Microbiol. 2014.

30. Choi YK, Goyal SM, Joo HS. Prevalence of swine influenza virus subtypes on swine farms in the United States. Arch Virol. 2002;147(6):1209-20.

31. Choi YK, Kim HS, Joo HS, Kim CJ. Isolation and genetic characterization of H1N2 subtype of influenza A virus from pigs in Korea. Journal of Bacteriology and Virology. 2003;33(1):93-9.

32. Choi YK, Goyal SM, Joo HS. Retrospective analysis of etiologic agents associated with respiratory diseases in pigs. The Canadian veterinary journal La revue veterinaire canadienne. 2003;44(9):735-7.

33. Choi C, Ha SK, Chae C. Detection and isolation of H1N1 influenza virus from pigs in Korea. Vet Rec. 2004;154(9):274-5.

34. Choi YK, Nguyen TD, Ozaki H, Webby RJ, Puthavathana P, Buranathal C, et al. Studies of H5N1 influenza virus infection of pigs by using viruses isolated in Vietnam and Thailand in 2004. J Virol. 2005;79(16):10821-5.

35. Choi MJ, Torremorell M, Bender JB, Smith K, Boxrud D, Ertl JR, et al. Live animal markets in Minnesota: a potential source for emergence of novel influenza A viruses and interspecies transmission. Clin Infect Dis. 2015.

36. Ciacci-Zanella JR, Schaefer R, Gava D, Haach V, Cantao ME, Coldebella A. Influenza A virus infection in Brazilian swine herds following the introduction of pandemic 2009 H1N1. Vet Microbiol. 2015.

37. Cong Y, Wang G, Guan Z, Chang S, Zhang Q, Yang G, et al. Reassortant between human-Like H3N2 and avian H5 subtype influenza A viruses in pigs: a potential public health risk. PLoS One. 2010;5(9):e12591.

38. Conlan JV, Vongxay K, Jarman RG, Gibbons RV, Lunt RA, Fenwick S, et al. Serologic Study of Pig-Associated Viral Zoonoses in Laos. Am J Trop Med Hyg. 2012;86(6):1077-84.

39. Corzo CA, Gramer M, Kuhn M, Mohr M, Morrison R. Observations regarding influenza A virus shedding in a swine breeding farm after mass vaccination. Journal of Swine Health and Production. 2012;20(6):283-9.

40. Corzo CA, Culhane M, Juleen K, Stigger-Rosser E, Ducatez MF, Webby RJ, et al. Active surveillance for influenza A virus among swine, midwestern United States, 2009-2011. Emerg Infect Dis. 2013;19(6):954-60.

41. Corzo CA, Morrison RB, Fitzpatrick AM, Culhane MR. Risk factors for detecting influenza A virus in growing pigs. Journal of Swine Health and Production. 2014;22(4):176-84.

42. Couacy-Hymann E, Kouakou VA, Aplogan GL, Awoume F, Kouakou CK, Kakpo L, et al. Surveillance for influenza viruses in poultry and Swine, west Africa, 2006-2008. Emerg Infect Dis. 2012;18(9):1446-52.

43. Cuong NV, Carrique-Mas J, Thu HTV, Hien ND, Hoa NT, Nguyet LA, et al. Serological and virological surveillance for porcine reproductive and respiratory syndrome virus, porcine circovirus type 2, and influenza A viruses among smallholder swine farms of the Mekong Delta, Vietnam. Journal of Swine Health and Production. 2014;22(5):224-31.

44. Davidson I, Al-Touri A, Raibshtein I, Hadani Y, Bombarov V, Yadin H, et al. Seroepidemiology Survey and Isolation of Swine Influenza Viruses from Subclinical Infections in Israel During the Years 2009-2011. Israel Journal of Veterinary Medicine. 2014;69(2):62-7.

45. Dias AS, Costa EA, Rajao DS, Guedes RM, Ciacci Zanella JR, Lobato ZI. Distribution of antibodies against influenza virus in pigs from farrow-to-finish farms in Minas Gerais state, Brazil. Influenza Other Respir Viruses. 2015.

46. Diaz A, Perez A, Sreevatsan S, Davies P, Culhane M, Torremorell M. Association between Influenza A Virus Infection and Pigs Subpopulations in Endemically Infected Breeding Herds. PLoS One. 2015;10(6):e0129213.

47. Dibarbora M, Cappuccio J, Olivera V, Quiroga M, Machuca M, Perfumo C, et al. Swine influenza: clinical, serological, pathological, and virological cross-sectional studies in nine farms in Argentina. Influenza Other Respir Viruses. 2013;7 Suppl 4:10-5.

48. El-Sayed A, Awad W, Fayed A, Hamann HP, Zschock M. Avian influenza prevalence in pigs, Egypt. Emerg Infect Dis. 2010;16(4):726-7.

49. El-Sayed A, Prince A, Fawzy A, Nadra E, Abdou MI, Omar L, et al. Sero-prevalence of avian influenza in animals and human in Egypt. Pakistan journal of biological sciences: PJBS. 2013;16(11):524-9.

50. Fablet C, Simon G, Dorenlor V, Eono F, Eveno E, Gorin S, et al. Different herd level factors associated with H1N1 or H1N2 influenza virus infections in fattening pigs. Prev Vet Med. 2013.

51. Fan X, Zhu H, Zhou B, Smith DK, Chen X, Lam TT, et al. Emergence and dissemination of a swine H3N2 reassortant influenza virus with 2009 pandemic H1N1 genes in pigs in China. J Virol. 2012;86(4):2375-8.

52. Forberg H, Hauge AG, Gjerset B, Hungnes O, Kilander A. Swine influenza in Norway: a distinct lineage of influenza A(H1N1)pdm09 virus. Influenza Other Respir Viruses. 2013;7 Suppl 4:21-6.

53. Foti M, Bottari T, Daidone A, Rinaldo D, De Leo F, Foti S, et al. Serological survey on Aujeszky's disease, swine influenza and porcine reproductive and respiratory syndrome virus infections in Italian pigs. Polish journal of veterinary sciences. 2008;11(4):323-5.

54. Fragaszy E, Ishola DA, Brown IH, Enstone J, Nguyen-Van-Tam JS, Simons R, et al. Increased risk of A(H1N1)pdm09 influenza infection in UK pig industry workers compared to a general population cohort. Influenza Other Respir Viruses. 2016;10(4):291-300.

55. Gjerset B, Er C, Lotvedt S, Jorgensen A, Hungnes O, Lium B, et al. Experiences after Twenty Months with Pandemic Influenza A (H1N1) 2009 Infection in the Naive Norwegian Pig Population. Influenza research and treatment. 2011;2011:206975.

56. Goto H, Takai M, Iguchi H, Benkele W, Ohta C, Yamamoto Y, et al. Antibody responses of swine to type A influenza viruses in the most recent several years. J Vet Med Sci. 1992;54(2):235-41.

57. Gray GC, Bender JB, Bridges CB, Daly RF, Krueger WS, Male MJ, et al. Influenza A(H1N1)pdm09 Virus among Healthy Show Pigs, United States. Emerg Infect Dis. 2012;18(9):1519-21.

58. Grontvedt CA, Er C, Gjerset B, Hauge AG, Brun E, Jorgensen A, et al. Influenza A(H1N1)pdm09 virus infection in Norwegian swine herds 2009/10: The risk of human to swine transmission. Prev Vet Med. 2013;110(3-4):429-34.

59. Groschup MH, Brun A, Haas B. Serological studies on the potential synergism of porcine reproductive and respiratory syndrome virus and influenza-, corona- and paramyxoviruses in the induction of respiratory symptoms in swine. Zentralblatt fur Veterinarmedizin Reihe B Journal of veterinary medicine Series B. 1993;40(9-10):681-9.

60. Gu FX, Qi WB, Su S, Chen JD, Qi HT, Zhu WJ, et al. Serological surveillance of swine influenza virus infection in seven cities located in Guangdong province, South China in 2010. Journal of Animal and Veterinary Advances. 2012;11(17):3068-72.

61. Guan Y, Shortridge KF, Krauss S, Li PH, Kawaoka Y, Webster RG. Emergence of avian H1N1 influenza viruses in pigs in China. J Virol. 1996;70(11):8041-6.

62. Guercio A, Purpari G, Conaldi PG, Pagano V, Moreno A, Giambruno P, et al. Pandemic influenza A/H1N1 virus in a swine farm house in Sicily, Italy. Journal of environmental biology / Academy of Environmental Biology, India. 2012;33(2):155-7.

63. Han JY, Park SJ, Kim HK, Rho S, Nguyen GV, Song D, et al. Identification of reassortant pandemic H1N1 influenza virus in Korean pigs. Journal of microbiology and biotechnology. 2012;22(5):699-707.

64. Hansen MS, Pors SE, Jensen HE, Bille-Hansen V, Bisgaard M, Flachs EM, et al. An investigation of the pathology and pathogens associated with porcine respiratory disease complex in Denmark. Journal of comparative pathology. 2010;143(2-3):120-31.

65. Harder TC, Grosse Beilage E, Lange E, Meiners C, Dohring S, Pesch S, et al. Expanded cocirculation of stable subtypes, emerging lineages, and new sporadic reassortants of porcine influenza viruses in swine populations in Northwest Germany. J Virol. 2013;87(19):10460-76.

66. He L, Zhao G, Zhong L, Liu Q, Duan Z, Gu M, et al. Isolation and characterization of two H5N1 influenza viruses from swine in Jiangsu Province of China. Arch Virol. 2013;158(12):2531-41.

67. Hofshagen M, Gjerset B, Er C, Tarpai A, Brun E, Dannevig B, et al. Pandemic influenza A(H1N1)v: human to pig transmission in Norway? Euro Surveill. 2009;14(45).

68. Holyoake PK, Kirkland PD, Davis RJ, Arzey KE, Watson J, Lunt RA, et al. The first identified case of pandemic H1N1 influenza in pigs in Australia. Aust Vet J. 2011;89(11):427-31.

69. Howard WA, Essen SC, Strugnell BW, Russell C, Barass L, Reid SM, et al. Reassortant Pandemic (H1N1) 2009 virus in pigs, United Kingdom. Emerg Infect Dis. 2011;17(6):1049-52.

70. Jacques-Simon R, Millien M, Flanagan JK, Shaw J, Morales P, Pinto J, et al. A field and laboratory investigation of viral diseases of swine in the Republic of Haiti. Journal of Swine Health and Production. 2013;21(3):130-8.

71. Jimenez LF, Nieto GR, Alfonso VV, Correa JJ. Association of swine influenza H1N1 pandemic virus (SIV-H1N1p) with porcine respiratory disease complex in sows from commercial pig farms in Colombia. Virologica Sinica. 2014;29(4):242-9.

72. Jo SK, Kim HS, Cho SW, Seo SH. Genetic and antigenic characterization of swine H1N2 influenza viruses isolated from Korean pigs. Journal of microbiology and biotechnology. 2007;17(5):868-72.

73. Jung T, Choi C, Chung HK, Kim J, Cho WS, Jung K, et al. Herd-level seroprevalence of swine-influenza virus in Korea. Prev Vet Med. 2002;53(4):311-4.

74. Jung K, Song DS. Evidence of the co-circulation of influenza H1N1, H1N2 and H3N2 viruses in the pig population of Korea. Vet Rec. 2007;161(3):104-5.

75. Jung K, Song DS, Kang BK, Oh JS, Park BK. Serologic surveillance of swine H1 and H3 and avian H5 and H9 influenza A virus infections in swine population in Korea. Prev Vet Med. 2007;79(2-4):294-303.

76. Kaplan BS, DeBeauchamp J, Stigger-Rosser E, Franks J, Crumpton JC, Turner J, et al. Influenza Virus Surveillance in Coordinated Swine Production Systems, United States. Emerg Infect Dis. 2015;21(10):1834-6.

77. Karlsson EA, Ciuoderis K, Freiden PJ, Seufzer B, Jones JC, Johnson J, et al. Prevalence and characterization of influenza viruses in diverse species in Los Llanos, Colombia. Emerging Microbes and Infections. 2013;2.

78. Katsuda K, Shirahata T, Kida H, Goto H. Antigenic and genetic analyses of the hemagglutinin of influenza viruses isolated from pigs in 1993. J Vet Med Sci. 1995;57(6):1023-7.

79. Katsuda K, Sato S, Shirahata T, Lindstrom S, Nerome R, Ishida M, et al. Antigenic and genetic characteristics of H1N1 human influenza virus isolated from pigs in Japan. J Gen Virol. 1995;76 ( Pt 5):1247-9.

80. Katsuda K, Sato S, Imai M, Shirahata T, Goto H. Prevalence of antibodies to type A influenza viruses in swine sera 1990-1994. J Vet Med Sci. 1995;57(4):773-5.

81. Killian ML, Swenson SL, Vincent AL, Landgraf JG, Shu B, Lindstrom S, et al. Simultaneous infection of pigs and people with triple-reassortant swine influenza virus H1N1 at a U.S. county fair. Zoonoses Public Health. 2013;60(3):196-201.

82. Kim JI, Lee I, Park S, Lee S, Hwang MW, Bae JY, et al. Phylogenetic analysis of a swine influenza A(H3N2) virus isolated in Korea in 2012. PLoS One. 2014;9(2):e88782.

83. Kirunda H, Erima B, Tumushabe A, Kiconco J, Tugume T, Mulei S, et al. Prevalence of influenza A viruses in livestock and free-living waterfowl in Uganda. BMC Vet Res. 2014;10:50.

84. Kobayashi M, Takayama I, Kageyama T, Tsukagoshi H, Saitoh M, Ishioka T, et al. Novel reassortant influenza A(H1N2) virus derived from A(H1N1)pdm09 virus isolated from swine, Japan, 2012. Emerg Infect Dis. 2013;19(12):1972-4.

85. Kong WL, Huang YM, Cao N, Qi HT, Huang LZ, Zhao MM, et al. Isolation and Phylogenetic Analysis of H1N1 Swine Influenza Virus from Sick Pigs in Southern China. Indian journal of virology : an official organ of Indian Virological Society. 2011;22(1):66-71.

86. Kong WL, Huang LZ, Cao N, Qi HT, Zhao MM, Guan SS, et al. Isolation and phylogenetic analysis of h9n2 swine influenza virus from sick pigs in Southern China in 2010. Journal of Animal and Veterinary Advances. 2011;10(18):2331-42.

87. Kowalczyk A, Markowska-Daniel I. Phylogenetic analysis of swine influenza viruses isolated in Poland. Polish journal of veterinary sciences. 2010;13(1):37-44.

88. Kwon TY, Lee SS, Kim CY, Shin JY, Sunwoo SY, Lyoo YS. Genetic characterization of H7N2 influenza virus isolated from pigs. Vet Microbiol. 2011;153(3-4):393-7.

89. Kyriakis CS, Brown IH, Foni E, Kuntz-Simon G, Maldonado J, Madec F, et al. Virological surveillance and preliminary antigenic characterization of influenza viruses in pigs in five European countries from 2006 to 2008. Zoonoses Public Health. 2011;58(2):93-101.

90. Kyriakis CS, Rose N, Foni E, Maldonado J, Loeffen WL, Madec F, et al. Influenza A virus infection dynamics in swine farms in Belgium, France, Italy and Spain, 2006-2008. Vet Microbiol. 2013.

91. Kyriakis CS, Papatsiros VG, Athanasiou LV, Valiakos G, Brown IH, Simon G, et al. Serological Evidence of Pandemic H1N1 Influenza Virus Infections in Greek Swine. Zoonoses Public Health. 2015.

92. Lange J, Groth M, Schlegel M, Krumbholz A, Wieczorek K, Ulrich R, et al. Reassortants of the pandemic (H1N1) 2009 virus and establishment of a novel porcine H1N2 influenza virus, lineage in Germany. Vet Microbiol. 2013;167(3-4):345-56.

93. Larison B, Njabo KY, Chasar A, Fuller T, Harrigan RJ, Smith TB. Spillover of pH1N1 to swine in Cameroon: an investigation of risk factors. BMC Vet Res. 2014;10:55.

94. Lee CS, Kang BK, Kim HK, Park SJ, Park BK, Jung K, et al. Phylogenetic analysis of swine influenza viruses recently isolated in Korea. Virus Genes. 2008;37(2):168-76.

95. Lee JH, Pascua PN, Song MS, Baek YH, Kim CJ, Choi HW, et al. Isolation and genetic characterization of H5N2 influenza viruses from pigs in Korea. J Virol. 2009;83(9):4205-15.

96. Lekcharoensuk P, Nanakorn J, Wajjwalku W, Webby R, Chumsing W. First whole genome characterization of swine influenza virus subtype H3N2 in Thailand. Vet Microbiol. 2010;145(3-4):230-44.

97. Li H, Yu K, Xin X, Yang H, Li Y, Qin Y, et al. Serological and virologic surveillance of swine influenza in China from 2000 to 2003. International Congress Series. 2004;1263(0):754-7.

98. Li S, Zhou Y, Zhao Y, Li W, Song W, Miao Z. Avian influenza H9N2 seroprevalence among pig population and pig farm staff in Shandong, China. Virol J. 2015;12:34.

99. Liu J, Bi Y, Qin K, Fu G, Yang J, Peng J, et al. Emergence of European avian influenza virus-like H1N1 swine influenza A viruses in China. J Clin Microbiol. 2009;47(8):2643-6.

100. Liu GH, Zhou DH, Cong W, Zhang XX, Shi XC, Danba C, et al. First report of seroprevalence of swine influenza A virus in Tibetan pigs in Tibet, China. Trop Anim Health Prod. 2014;46(1):257-9.

101. Loeffen WL, Kamp EM, Stockhofe-Zurwieden N, van Nieuwstadt AP, Bongers JH, Hunneman WA, et al. Survey of infectious agents involved in acute respiratory disease in finishing pigs. Vet Rec. 1999;145(5):123-9.

102. Lopez-Robles G, Montalvo-Corral M, Burgara-Estrella A, Hernandez J. Serological and molecular prevalence of swine influenza virus on farms in northwestern Mexico. Vet Microbiol. 2014;172(1-2):323-8.

103. Lopez-Soria S, Maldonado J, Riera P, Nofrarias M, Espinal A, Valero O, et al. Selected Swine viral pathogens in indoor pigs in Spain. Seroprevalence and farm-level characteristics. Transbound Emerg Dis. 2010;57(3):171-9.

104. Lutteke N, Sausy A, Black AP, Wildschutz F, Muller CP. Neutralizing antibodies against influenza A in pigs before and after the 2009 pandemic, Luxembourg. Vet Microbiol. 2014;169(1-2):96-101.

105. Ma W, Vincent AL, Gramer MR, Brockwell CB, Lager KM, Janke BH, et al. Identification of H2N3 influenza A viruses from swine in the United States. Proc Natl Acad Sci U S A. 2007;104(52):20949-54.

106. Ma W, Vincent AL, Lager KM, Janke BH, Henry SC, Rowland RR, et al. Identification and characterization of a highly virulent triple reassortant H1N1 swine influenza virus in the United States. Virus Genes. 2010;40(1):28-36.

107. Maes D, Deluyker H, Verdonck M, Castryck F, Miry C, Vrijens B, et al. Risk indicators for the seroprevalence of Mycoplasma hyopneumoniae, porcine influenza viruses and Aujeszky's disease virus in slaughter pigs from fattening pig herds. Zentralblatt fur Veterinarmedizin Reihe B Journal of veterinary medicine Series B. 1999;46(5):341-52.

108. Maes D, Deluyker H, Verdonck M, Castryck F, Miry C, Vrijens B, et al. Herd factors associated with the seroprevalences of four major respiratory pathogens in slaughter pigs from farrow-to-finish pig herds. Vet Res. 2000;31(3):313-27.

109. Maldonado J, Van Reeth K, Riera P, Sitja M, Saubi N, Espuna E, et al. Evidence of the concurrent circulation of H1N2, H1N1 and H3N2 influenza A viruses in densely populated pig areas in Spain. Vet J. 2006;172(2):377-81.

110. Markowska-Daniel I, Stankevicius A. Seroprevalence of antibodies against swine influenza virus in pigs of different age. Bulletin of the Veterinary Institute in Pulawy. 2005;49(1):3-7.

111. Markowska-Daniel I, Kwit K, Urbaniak K, Kowalczyk A. Serological evidence of co-circulation of different subtypes of swine influenza virus in Polish pig herds. Bulletin of the Veterinary Institute in Pulawy. 2012;56(4):425-9.

112. Mastin A, Alarcon P, Pfeiffer D, Wood J, Williamson S, Brown I, et al. Prevalence and risk factors for swine influenza virus infection in the English pig population. PLoS Curr. 2011;3:RRN1209.

113. Meemken D, Tangemann AH, Meermeier D, Gundlach S, Mischok D, Greiner M, et al. Establishment of serological herd profiles for zoonoses and production diseases in pigs by "meat juice multi-serology". Prev Vet Med. 2014;113(4):589-98.

114. Meiners C, Loesken S, Doehring S, Starick E, Pesch S, Maas A, et al. Field study on swine influenza virus (SIV) infection in weaner pigs and sows. Tierarztliche Praxis Ausgabe G, Grosstiere/Nutztiere. 2014;42(6):351-9.

115. Meseko CA, Odaibo GN, Olaleye DO. Detection and isolation of 2009 pandemic influenza A/H1N1 virus in commercial piggery, Lagos Nigeria. Vet Microbiol. 2014;168(1):197-201.

116. Meseko CA, Odurinde O, Olaniran O. Influenza infections in live pig market, Nigeria. International Journal of Infectious Diseases. 2014;21, Supplement 1(0):267.

117. Meyns T, Van Steelant J, Rolly E, Dewulf J, Haesebrouck F, Maes D. A cross-sectional study of risk factors associated with pulmonary lesions in pigs at slaughter. The Veterinary Journal. 2011;187(3):388-92.

118. Monger VR, Stegeman JA, Koop G, Dukpa K, Tenzin T, Loeffen WL. Seroprevalence and associated risk factors of important pig viral diseases in Bhutan. Prev Vet Med. 2014.

119. Mores MAZ, Oliveira JX, Rebelatto R, Klein CS, Barcellos DEN, Coldebella A, et al. Pathological and microbiological aspects of respiratory disease in fattening pigs in Brazil. Pesquisa Veterinaria Brasileira. 2015;35(8):725-33.

120. Nakharuthai C, Boonsoongnern A, Poolperm P, Wajjwalku W, Urairong K, Chumsing W, et al. Occurrence of swine influenza virus infection in swine with porcine respiratory disease complex. The Southeast Asian journal of tropical medicine and public health. 2008;39(6):1045-53.

121. Nathues H, Chang YM, Wieland B, Rechter G, Spergser J, Rosengarten R, et al. Herd-level risk factors for the seropositivity to Mycoplasma hyopneumoniae and the occurrence of enzootic pneumonia among fattening pigs in areas of endemic infection and high pig density. Transbound Emerg Dis. 2014;61(4):316-28.

122. Nelson MI, Schaefer R, Gava D, Cantao ME, Ciacci-Zanella JR. Influenza A Viruses of Human Origin in Swine, Brazil. Emerg Infect Dis. 2015;21(8):1339-47.

123. Nelson M, Culhane MR, Rovira A, Torremorell M, Guerrero P, Norambuena J. Novel Human-like Influenza A Viruses Circulate in Swine in Mexico and Chile. PLoS Curr. 2015;7.

124. Netrabukkana P, Cappelle J, Trevennec C, Roger F, Goutard F, Buchy P, et al. Epidemiological Analysis of Influenza A Infection in Cambodian Pigs and Recommendations for Surveillance Strategies. Transbound Emerg Dis. 2014.

125. Ngo LT, Hiromoto Y, Pham VP, Le HT, Nguyen HT, Le VT, et al. Isolation of novel triple-reassortant swine H3N2 influenza viruses possessing the hemagglutinin and neuraminidase genes of a seasonal influenza virus in Vietnam in 2010. Influenza Other Respir Viruses. 2012;6(1):6-10.

126. Nidom CA, Takano R, Yamada S, Sakai-Tagawa Y, Daulay S, Aswadi D, et al. Influenza A (H5N1) viruses from pigs, Indonesia. Emerg Infect Dis. 2010;16(10):1515-23.

127. Ninomiya A, Takada A, Okazaki K, Shortridge KF, Kida H. Seroepidemiological evidence of avian H4, H5, and H9 influenza A virus transmission to pigs in southeastern China. Vet Microbiol. 2002;88(2):107-14.

128. Njabo KY, Fuller TL, Chasar A, Pollinger JP, Cattoli G, Terregino C, et al. Pandemic A/H1N1/2009 influenza virus in swine, Cameroon, 2010. Vet Microbiol. 2012;156(1-2):189-92.

129. Nokireki T, Laine T, London L, Ikonen N, Huovilainen A. The first detection of influenza in the Finnish pig population: a retrospective study. Acta veterinaria Scandinavica. 2013;55:69.

130. Nonthabenjawan N, Chanvatik S, Chaiyawong S, Jairak W, Boonyapisusopha S, Tuanudom R, et al. Genetic diversity of swine influenza viruses in Thai swine farms, 2011-2014. Virus Genes. 2015;50(2):221-30.

131. Nowotny N, Mostl K, Maderbacher R, Odorfer G, Schuh M. Serological studies in Austrian fattening pigs with respiratory disorders. Acta veterinaria Hungarica. 1994;42(2-3):377-9.

132. Oladipo EK, Adeniji JA, Fagbam AH. Isolation of influenza A and B viruses from pigs at Bodija Abattoir, Ibadan, Nigeria. Global Veterinaria. 2013;11(2):252-7.

133. Olsen CW, Carey S, Hinshaw L, Karasin AI. Virologic and serologic surveillance for human, swine and avian influenza virus infections among pigs in the north-central United States. Arch Virol. 2000;145(7):1399-419.

134. Ouchi A, Nerome K, Kanegae Y, Ishida M, Nerome R, Hayashi K, et al. Large outbreak of swine influenza in southern Japan caused by reassortant (H1N2) influenza viruses: its epizootic background and characterization of the causative viruses. J Gen Virol. 1996;77 ( Pt 8):1751-9.

135. Ozawa M, Matsuu A, Yonezawa K, Igarashi M, Okuya K, Kawabata T, et al. Efficient isolation of Swine influenza viruses by age-targeted specimen collection. J Clin Microbiol. 2015;53(4):1331-8.

136. Palzer A, Ritzmann M, Wolf G, Heinritzi K. Associations between pathogens in healthy pigs and pigs with pneumonia. Vet Rec. 2008;162(9):267-71.

137. Papatsiros VG, Athanasiou LV, Psalla D, Petridou E, Maragkakis GG, Papatsas I, et al. Erythema Multiforme Associated with Respiratory Disease in a Commercial Breeding Pig Herd. Viral Immunol. 2015;28(8):464-71.

138. Pascua PN, Song MS, Lee JH, Choi HW, Han JH, Kim JH, et al. Seroprevalence and genetic evolutions of swine influenza viruses under vaccination pressure in Korean swine herds. Virus Res. 2008;138(1-2):43-9.

139. Pascua PN, Lim GJ, Kwon HI, Park SJ, Kim EH, Song MS, et al. Emergence of H3N2pM-like and novel reassortant H3N1 swine viruses possessing segments derived from the A (H1N1)pdm09 influenza virus, Korea. Influenza Other Respir Viruses. 2013;7(6):1283-91.

140. Peiris JS, Guan Y, Markwell D, Ghose P, Webster RG, Shortridge KF. Cocirculation of avian H9N2 and contemporary "human" H3N2 influenza A viruses in pigs in southeastern China: potential for genetic reassortment? J Virol. 2001;75(20):9679-86.

141. Pereda A, Cappuccio J, Quiroga MA, Baumeister E, Insarralde L, Ibar M, et al. Pandemic (H1N1) 2009 outbreak on pig farm, Argentina. Emerg Infect Dis. 2010;16(2):304-7.

142. Pereda A, Rimondi A, Cappuccio J, Sanguinetti R, Angel M, Ye J, et al. Evidence of reassortment of pandemic H1N1 influenza virus in swine in Argentina: are we facing the expansion of potential epicenters of influenza emergence? Influenza Other Respir Viruses. 2011;5(6):409-12.

143. Perera HK, Wickramasinghe G, Cheung CL, Nishiura H, Smith DK, Poon LL, et al. Swine influenza in Sri Lanka. Emerg Infect Dis. 2013;19(3):481-4.

144. Perera HK, Vijaykrishna D, Premarathna AG, Jayamaha CJ, Wickramasinghe G, Cheung CL, et al. Molecular epidemiology of influenza A(H1N1)pdm09 virus among humans and swine, Sri Lanka. Emerg Infect Dis. 2014;20(12):2080-4.

145. Perez LJ, Perera CL, Vega A, Frias MT, Rouseaux D, Ganges L, et al. Isolation and complete genomic characterization of pandemic H1N1/2009 influenza viruses from Cuban swine herds. Res Vet Sci. 2013;94(3):781-8.

146. Perez LJ, Perera CL, Coronado L, Rios L, Vega A, Frias MT, et al. Molecular epidemiology study of swine influenza virus revealing a reassorted virus H1N1 in swine farms in Cuba. Prev Vet Med. 2015;119(3-4):172-8.

147. Pineyro PE, Baumeister E, Cappuccio JA, Machuca MA, Quiroga MA, Tedoroff T, et al. [Seroprevalence of the swine influenza virus in fattening pigs in Argentina in the 2002 season: evaluation by hemagglutination-inhibition and ELISA tests]. Revista Argentina de microbiologia. 2010;42(2):98-101.

148. Poljak Z, Friendship RM, Carman S, McNab WB, Dewey CE. Investigation of exposure to swine influenza viruses in Ontario (Canada) finisher herds in 2004 and 2005. Prev Vet Med. 2008;83(1):24-40.

149. Poljak Z, Dewey CE, Martin SW, Christensen J, Carman S, Friendship RM. Prevalence of and risk factors for influenza in southern Ontario swine herds in 2001 and 2003. Can J Vet Res. 2008;72(1):7-17.

150. Poonsuk S, Sangthong P, Petcharat N, Lekcharoensuk P. Genesis and genetic constellations of swine influenza viruses in Thailand. Vet Microbiol. 2013;167(3-4):314-26.

151. Pospíšil Z, Lány P, Tůmová B, Buchta J, Zendulková D, Číhal P. Swine influenza surveillance and the impact of human influenza epidemics on pig herds in the Czech Republic. Acta Veterinaria Brno. 2001;70(3):327-32.

152. Pozzi SP, Aborali G, Cordioli P, Rosner A. Investigation of swine influenza sub-types H1N1, H32, N1N2 in pigs population in Israel (2002-2009). Israel Journal of Veterinary Medicine. 2010;65(1):11-4.

153. Qi X, Pan Y, Qin Y, Zu R, Tang F, Zhou M, et al. Molecular characterization of avian-like H1N1 swine influenza a viruses isolated in Eastern China, 2011. Virologica Sinica. 2012;27(5):292-8.

154. Qiao C, Liu L, Yang H, Chen Y, Xu H, Chen H. Novel triple reassortant H1N2 influenza viruses bearing six internal genes of the pandemic 2009/H1N1 influenza virus were detected in pigs in China. J Clin Virol. 2014;61(4):529-34.

155. Rajao DS, Couto DH, Gasparini MR, Costa ATR, Reis JKP, Lobato ZIP, et al. Diagnosis and clinic-pathological findings of influenza virus infection in Brazilian pigs. Pesquisa Veterinaria Brasileira. 2013;33(1):30-6.

156. Rajao DS, Alves F, Del Puerto HL, Braz GF, Oliveira FG, Ciacci-Zanella JR, et al. Serological evidence of swine influenza in Brazil. Influenza Other Respir Viruses. 2013;7(2):109-12.

157. Rammohan L, Xue L, Wang C, Chittick W, Ganesan S, Ramamoorthy S. Increased prevalence of torque teno viruses in porcine respiratory disease complex affected pigs. Vet Microbiol. 2012;157(1-2):61-8.

158. Regula G, Lichtensteiger CA, Mateus-Pinilla NE, Scherba G, Miller GY, Weigel RM. Comparison of serologic testing and slaughter evaluation for assessing the effects of subclinical infection on growth in pigs. J Am Vet Med Assoc. 2000;217(6):888-95.

159. Regula G, Scherba G, Mateus-Pinilla NE, Lichtensteiger CA, Miller GY, Weigel RM. The impact of endemic porcine reproductive and respiratory syndrome virus and other pathogens on reproductive performance in swine. Journal of Swine Health and Production. 2003;11(1):13-8.

160. Richards S, Glazier M, House J, Pontones P, Metcalf D, Marsh B, et al. Notes from the field: Outbreak of influenza A (H3N2) virus among persons and swine at a county fair - Indiana, July 2012. Morbidity and Mortality Weekly Report. 2012;61(29):561.

161. Rith S, Netrabukkana P, Sorn S, Mumford E, Mey C, Holl D, et al. Serologic evidence of human influenza virus infections in swine populations, Cambodia. Influenza Other Respir Viruses. 2013;7(3):271-9.

162. Saavedra-Montanez M, Carrera-Aguirre V, Castillo-Juarez H, Rivera-Benitez F, Rosas-Estrada K, Pulido-Camarillo E, et al. Retrospective serological survey of influenza viruses in backyard pigs from Mexico City. Influenza Other Respir Viruses. 2013;7(5):827-32.

163. Sabale SS, Pawar SD, More BK, Mishra AC. Seroprevalence of pandemic influenza H1N1 (2009) & seasonal influenza viruses in pigs in Maharashtra & Gujarat States, India, 2011. The Indian journal of medical research. 2013;138:267-9.

164. SACCVS. First cases of pandemic H1N1/09 influenza in Scottish pigs. Vet Rec. 2010;166(18):548-51.

165. Saito T, Takemae N, Abe H, Uchida Y. Swine influenza surveillance in the southeast Asia. J Disaster Res. 2014;9(5):839-41.

166. Schaefer R, Zanella JRC, Brentano L, Vincen AL, Ritterbusc GA, Silveira S, et al. Isolation and characterization of a pandemic H1N1 influenza virus in pigs in Brazil. Pesquisa Veterinaria Brasileira. 2011;31(9):761-7.

167. Schaefer R, Rech RR, Gava D, Cantao ME, da Silva MC, Silveira S, et al. A human-like H1N2 influenza virus detected during an outbreak of acute respiratory disease in swine in Brazil. Arch Virol. 2014.

168. Schmidt C, Cibulski SP, Andrade CP, Teixeira TF, Varela AP, Scheffer CM, et al. Swine Influenza Virus and Association with the Porcine Respiratory Disease Complex in Pig Farms in Southern Brazil. J Virol. 2015.

169. Shieh HK, Chang PC, Chen TH, Li KP, Chan CH. Surveillance of avian and swine influenza in the swine population in Taiwan, 2004. J Microbiol Immunol Infect. 2008;41(3):231-42.

170. Shimada Si, Ohtsuka T, Tanaka M, Mimura M, Shinohara M, Uchida K, et al. Existence of reassortant A (H1N2) swine influenza viruses in Saitama Prefecture, Japan. International Congress Series. 2004;1263(0):749-53.

171. Shin JY, Song MS, Lee EH, Lee YM, Kim SY, Kim HK, et al. Isolation and characterization of novel H3N1 swine influenza viruses from pigs with respiratory diseases in Korea. J Clin Microbiol. 2006;44(11):3923-7.

172. Simon-Grife M, Martin-Valls GE, Vilar MJ, Garcia-Bocanegra I, Mora M, Martin M, et al. Seroprevalence and risk factors of swine influenza in Spain. Vet Microbiol. 2011;149(1-2):56-63.

173. Snoeck CJ, Abiola OJ, Sausy A, Okwen MP, Olubayo AG, Owoade AA, et al. Serological evidence of pandemic (H1N1) 2009 virus in pigs, West and Central Africa. Vet Microbiol. 2015;176(1-2):165-71.

174. Song DS, Lee JY, Oh JS, Lyoo KS, Yoon KJ, Park YH, et al. Isolation of H3N2 swine influenza virus in South Korea. J Vet Diagn Invest. 2003;15(1):30-4.

175. Song MS, Lee JH, Pascua PN, Baek YH, Kwon HI, Park KJ, et al. Evidence of human-to-swine transmission of the pandemic (H1N1) 2009 influenza virus in South Korea. J Clin Microbiol. 2010;48(9):3204-11.

176. Song XH, Xiao H, Huang Y, Fu G, Jiang B, Kitamura Y, et al. Serological surveillance of influenza A virus infection in swine populations in Fujian province, China: no evidence of naturally occurring H5N1 infection in pigs. Zoonoses Public Health. 2010;57(4):291-8.

177. Sreta D, Tantawet S, Na Ayudhya SN, Thontiravong A, Wongphatcharachai M, Lapkuntod J, et al. Pandemic (H1N1) 2009 virus on commercial swine farm, Thailand. Emerg Infect Dis. 2010;16(10):1587-90.

178. Sreta D, Jittimanee S, Charoenvisal N, Amonsin A, Kitikoon P, Thanawongnuwech R. Retrospective swine influenza serological surveillance in the four highest pig density provinces of Thailand before the introduction of the 2009 pandemic Influenza A virus subtype H1N1 using various antibody detection assays. J Vet Diagn Invest. 2013;25(1):45-53.

179. Starick E, Lange E, Fereidouni S, Bunzenthal C, Hoveler R, Kuczka A, et al. Reassorted pandemic (H1N1) 2009 influenza A virus discovered from pigs in Germany. J Gen Virol. 2011;92(Pt 5):1184-8.

180. Su S, Qi W, Chen J, Zhu W, Huang Z, Xie J, et al. Seroepidemiological evidence of avian influenza A virus transmission to pigs in southern China. J Clin Microbiol. 2013;51(2):601-2.

181. Sun Y-F, Wang X-H, Li X-L, Zhang L, Li H-H, Lu C, et al. Novel triple-reassortant H1N1 swine influenza viruses in pigs in Tianjin, Northern China. Vet Microbiol. 2016;183:85-91.

182. Suriya R, Hassan L, Omar AR, Aini I, Tan CG, Lim YS, et al. Seroprevalence and risk factors for influenza A viruses in pigs in Peninsular Malaysia. Zoonoses Public Health. 2008;55(7):342-51.

183. Tinoco YO, Montgomery JM, Kasper MR, Nelson MI, Razuri H, Guezala MC, et al. Transmission dynamics of pandemic influenza A(H1N1)pdm09 virus in humans and swine in backyard farms in Tumbes, Peru. Influenza Other Respir Viruses. 2016;10(1):47-56.

184. Trevennec K, Mortier F, Lyazrhi F, Huong HT, Chevalier V, Roger F. Swine influenza in Vietnam: preliminary results of epidemiological studies. Influenza Other Respi Viruses. 2011;5:71-3.

185. Trevennec K, Grosbois V, Roger F, Ho TH, Berthouly-Salazar C, Chevalier V. Evidence for freedom from swine influenza in a remote area of Northern Vietnam. Acta Trop. 2012;122(1):160-3.

186. Trevennec K, Leger L, Lyazrhi F, Baudon E, Cheung CY, Roger F, et al. Transmission of pandemic influenza H1N1 (2009) in Vietnamese swine in 2009-2010. Influenza Other Respir Viruses. 2012;6(5):348-57.

187. Tu J, Zhou H, Jiang T, Li C, Zhang A, Guo X, et al. Isolation and molecular characterization of equine H3N8 influenza viruses from pigs in China. Arch Virol. 2009;154(5):887-90.

188. Valheim M, Gamlem H, Gjerset B, Germundsson A, Lium B. Pathological Findings and Distribution of Pandemic Influenza A (H1N1) 2009 Virus in Lungs from Naturally Infected Fattening Pigs in Norway. Influenza research and treatment. 2011;2011:565787.

189. Van Reeth K, Brown IH, Pensaert M. Isolations of H1N2 influenza A virus from pigs in Belgium. Vet Rec. 2000;146(20):588-9.

190. Van Reeth K, Brown IH, Durrwald R, Foni E, Labarque G, Lenihan P, et al. Seroprevalence of H1N1, H3N2 and H1N2 influenza viruses in pigs in seven European countries in 2002-2003. Influenza Other Respir Viruses. 2008;2(3):99-105.

191. Vijaykrishna D, Smith GJ, Pybus OG, Zhu H, Bhatt S, Poon LL, et al. Long-term evolution and transmission dynamics of swine influenza A virus. Nature. 2011;473(7348):519-22.

192. Wang JY, Ren JJ, Qiu YH, Liu HJ. Complete Genome Sequences of Six Avian-Like H1N1 Swine Influenza Viruses from Northwestern China. Genome announcements. 2013;1(1).

193. Wang J, Wu M, Hong W, Fan X, Chen R, Zheng Z, et al. Infectivity and Transmissibility of Avian H9N2 Influenza Viruses in Pigs. J Virol. 2016.

194. Watanabe TTN, de Almeida LL, Wouters F, Wouters ATB, Zlotowski P, Driemeier D. Histopathological and immunohistochemical findings of swine with spontaneous influenza a infection in Brazil, 2009-2010. Pesquisa Veterinaria Brasileira. 2012;32(11):1148-54.

195. Webby RJ, Swenson SL, Krauss SL, Gerrish PJ, Goyal SM, Webster RG. Evolution of swine H3N2 influenza viruses in the United States. J Virol. 2000;74(18):8243-51.

196. Weingartl HM, Berhane Y, Hisanaga T, Neufeld J, Kehler H, Emburry-Hyatt C, et al. Genetic and pathobiologic characterization of pandemic H1N1 2009 influenza viruses from a naturally infected swine herd. J Virol. 2010;84(5):2245-56.

197. Wellenberg GJ, Bouwkamp FT, Wolf PJ, Swart WA, Mombarg MJ, de Gee AL. A study on the severity and relevance of porcine circovirus type 2 infections in Dutch fattening pigs with respiratory diseases. Vet Microbiol. 2010;142(3-4):217-24.

198. Weller CB, Cadmus KJ, Ehrhart EJ, Powers BE, Pabilonia KL. Detection and isolation of Influenza A virus subtype H1N1 from a small backyard swine herd in Colorado. J Vet Diagn Invest. 2013;25(6):782-4.

199. Welsh MD, Baird PM, Guelbenzu-Gonzalo MP, Hanna A, Reid SM, Essen S, et al. Initial incursion of pandemic (H1N1) 2009 influenza A virus into European pigs. Vet Rec. 2010;166(21):642-5.

200. Williamson SM, Tucker AW, McCrone IS, Bidewell CA, Brons N, Habernoll H, et al. Descriptive clinical and epidemiological characteristics of influenza A H1N1 2009 virus infections in pigs in England. Vet Rec. 2012;171(11):271.

201. Wu R, Liu Z, Liang W, Yang K, Deng J, Duan Z, et al. Serological and virological surveillance of swine H1N1 and H3N2 influenza virus infection in two farms located in Hubei province, central China. Zoonoses Public Health. 2011;58(7):508-13.

202. Xu C, Fan W, Wei R, Zhao H. Isolation and identification of swine influenza recombinant A/Swine/Shandong/1/2003(H9N2) virus. Microbes and infection / Institut Pasteur. 2004;6(10):919-25.

203. Xu C, Zhu Q, Yang H, Zhang X, Qiao C, Chen Y, et al. Two genotypes of H1N2 swine influenza viruses appeared among pigs in China. J Clin Virol. 2009;46(2):192-5.

204. Yaeger MJ, Karriker LA, Layman L, Halbur PG, Huber GH, Van Hulzen K. Survey of disease pressures in twenty-six niche herds in the midwestern United States. Journal of Swine Health and Production. 2009;17(5):256-63.

205. Yan JH, Xiong Y, Yi CH, Sun XX, He QS, Fu W, et al. Pandemic (H1N1) 2009 virus circulating in pigs, Guangxi, China. Emerg Infect Dis. 2012;18(2):357-9.

206. Yang H, Qiao C, Tang X, Chen Y, Xin X, Chen H. Human infection from avian-like influenza A (H1N1) viruses in pigs, China. Emerg Infect Dis. 2012;18(7):1144-6.

207. Yao Y, Zhang GH, Liu WJ, Chen TQ, Sun L. [Genome sequence analysis of an H3N2 subtype swine influenza virus isolated from Guangdong province in China]. Wei sheng wu xue bao = Acta microbiologica Sinica. 2007;47(5):805-9.

208. Yu H, Zhou YJ, Li GX, Zhang GH, Liu HL, Yan LP, et al. Further evidence for infection of pigs with human-like H1N1 influenza viruses in China. Virus Res. 2009;140(1-2):85-90.

209. Yu H, Zhang PC, Zhou YJ, Li GX, Pan J, Yan LP, et al. Isolation and genetic characterization of avian-like H1N1 and novel ressortant H1N2 influenza viruses from pigs in China. Biochemical and biophysical research communications. 2009;386(2):278-83.

210. Yuan Z, Zhu W, Chen Y, Zhou P, Cao Z, Xie J, et al. Serological surveillance of H5 and H9 avian influenza A viral infections among pigs in Southern China. Microbial pathogenesis. 2013;64:39-42.

211. Zhang G, Kong W, Qi W, Long LP, Cao Z, Huang L, et al. Identification of an H6N6 swine influenza virus in southern China. Infect Genet Evol. 2011;11(5):1174-7.

212. Zhao G, Pan J, Gu X, Lu X, Li Q, Zhu J, et al. Isolation and phylogenetic analysis of avian-origin European H1N1 swine influenza viruses in Jiangsu, China. Virus Genes. 2012;44(2):295-300.

213. Zhao G, Fan Q, Zhong L, Li Y, Liu W, Liu X, et al. Isolation and phylogenetic analysis of pandemic H1N1/09 influenza virus from swine in Jiangsu province of China. Res Vet Sci. 2012;93(1):125-32.

214. Zhao G, Chen C, Huang J, Wang Y, Peng D, Liu X. Characterisation of one H6N6 influenza virus isolated from swine in China. Res Vet Sci. 2013;95(2):434-6.

215. Zhou N, He S, Zhang T, Zou W, Shu L, Sharp GB, et al. Influenza infection in humans and pigs in southeastern China. Arch Virol. 1996;141(3-4):649-61.

216. Zhou H, Cao Z, Tan L, Fu X, Lu G, Qi W, et al. Avian-like A (H1N1) swine influenza virus antibodies among swine farm residents and pigs in southern China. Japanese journal of infectious diseases. 2014;67(3):184-90.

217. Zhou P, Hong M, Merrill MM, He H, Sun L, Zhang G. Serological report of influenza A (H7N9) infections among pigs in Southern China. BMC Vet Res. 2014;10(1):203.
