# Supplementary material for: Epidemiological features of influenza circulation in swine populations: A systematic review and meta-analysis
Source: PLoS One. 2017 Jun 7;12(6):e0179044. doi: 10.1371/journal.pone.0179044 (PMC5462427; doi:10.1371/journal.pone.0179044)
Supplement: S1 Table — (DOCX) [file pone.0179044.s006.docx]

**Epidemiological features of influenza circulation in swine populations: a systematic review and meta-analysis**

Eugénie Baudon, Marisa Peyre, Malik Peiris, Benjamin J. Cowling

S1 Table. Risk factors associated with influenza circulation in studies carried out on or after 1990

References can be found in S1 References. *Values from univariate analyses (no multivariate analysis done)

F=Farm, S=Slaughterhouse, A=Agricultural fairs; Pos=positively associated, Neg=negatively associated.

| **Category** | **Detail** | **OR (Estimate)** | **OR 95% CI (Estimate)** | **p-value** | **Subtype** | **Pre-mise** | **Country** | **Refe-rence** |
| --- | --- | --- | --- | --- | --- | --- | --- | --- |
| **Swine population** | number of other pig herds >2 in the vicinity (2km) | 3.2 | 1.4-7.6 | 0.01 | H1N1 | F | France | 50 |
|  | number of other pig herds >2 in the vicinity (2km) | 3.5 | 1.5-8.1 | <0.01 | H1N2 | F | France | 50 |
|  | number of pig herds in the municipality (increase of one herd/km2) | 1.98 | 1.11-3.55 | 0.022 | H1N1 | F | Belgium | 108 |
|  | number of pig herds in the municipality (increase of one herd/km2) | 1.46 | 1.07-2.01 | 0.019 | H3N2 | F | Belgium | 108 |
|  | reported distance to the nearest barn | (-0.34) | (-0.69,0.02) | 0.02 | H1N1 | F | Canada | 148 |
|  | distance to nearest pig barn (<1 & 1-3km) | 7.32 & 10.18 | 1.49–55.15 & 1.51-204.38 | 0.01* |  | F | Canada | 149 |
|  | pig farm density (0.1 farms/km2) | 1.41 | 1.00-2.04 | 0.02 |  | F | Canada | 149 |
|  | pig farm density (0.1 farms/km2) (different census data) | 32.46, 317.35 | 3.60-1422.26, 13.87->9999 | <0.01* |  | F | Canada | 149 |
|  | far location to another farm | 0.06 | 0.02-0.16 | 0.00 | H3N2 | F | Malaysia | 182 |
|  | number of pigs in the municipality (increase of 500 pigs/km2) | 1.35 | - | 0.025 | H3N2 | F | Belgium | 107 |
|  | pig density | (-0.0350) | - | 9.03E-05 | H3N2 | F,S | Cambodia | 124 |
|  | pig density (100 pigs/km2) (different census data) | 1.02, 1.05 | 1.01-1.05, 1.02-1.09 | 0.02, <0.01* |  | F | Canada | 149 |
|  | number of pigs at a fair (increase 20 pigs) | 1.012 | 1.002-1.026 | 0.012 |  | A | USA | 16 |
|  | number of pigs per pen in the post-weaning room >28 | 3.2 | 1.2-8.6 | 0.02 | H1N1 | F | France | 50 |
|  | number of pigs in the fattening room >110 | 2.5 | 1.1-5.9 | 0.03 | H1N2 | F | France | 50 |
|  | herd size | 1.01 | 1-1.02 | 0.009 | H1N1pdm09 | F | Norway | 58 |
|  | number of fattening pigs per pen (increase of one pig/pen) | 1.2 | 1.03-1.40 | 0.02 | H3N2 | F | Belgium | 108 |
|  | more than 18 finishers per water space | 5.22 | 1.57-17.43 | 0.01 |  | F | UK | 112 |
|  | number of pigs in a barn (100) | (0.10) | (0.01,0.2) | 0.01 | H1N1 | F | Canada | 148 |
|  | number of sows | curvilinear | - | 0.05* |  | F | Canada | 149 |
|  | increase in number of finisher pigs by 1000 | 4.44 | 1.90-13.07 | <0.01 |  | F | Canada | 149 |
|  | farm size >5000 pigs | 4.97 | 1.74-14.14 | 0 | H1N1 | F | Malaysia | 182 |
|  | farm size >5000 pigs | 9.16 | 3.23-25.98 | 0 | H3N2 | F | Malaysia | 182 |
|  | Government breeding farms vs village backyard farm | pos | - | -* | H1N1 | F | Bhutan | 118 |
|  | new gilts compared to gilts | 7.9 | 1.4-43.9 | <0.05 |  | F | USA | 46 |
|  | piglets compared to gilts | 4.4 | 1.1-17.1 | <0.05 |  | F | USA | 46 |
|  | piglets compared to gilts | 1.3 | 1.1-1.6 | 0.004 |  | F | USA | 76 |
|  | pigs in multiplier farms vs breed-to-wean farms | 0.7 | 0.5-0.9 | 0.011 |  | F | USA | 76 |
|  | pigs in gilt development units vs breed-to-wean farms | 1.6 | 1.2-2.1 | <0.001 |  | F | USA | 76 |
| **Farm management** | purchase from ≥2 herds | 9.68 | - | 0.023 | H3N2 | F | Belgium | 107 |
|  | no purchase of pigs | 0.33 | 0.16-0.60 | 0.00 | H1N1 | F | Malaysia | 182 |
|  | no purchase of pigs | 0.23 | 0.11-0.44 | 0.00 | H3N2 | F | Malaysia | 182 |
|  | external source of gilts | 4.62 | 1.14-20.91 | 0.03* |  | F | Canada | 149 |
|  | parity of the sow | pos | - | - |  | F | Canada | 149 |
|  | sow replacement rate | 1.02, 1.04, 1.07 | 1.01-1.04, 1.01-1.07, 1.02-1.12 | 0.007, 0.005, NA |  | F | Spain | 172 |
|  | fattening farm only | 0.35 | 0.17-0.70 | <0.01 |  | F | Vietnam | 186 |
|  | finisher only | 0.11 | 0.01-0.62 | <0.01 |  | F | Canada | 149 |
|  | farm type (farrow-to-finish and nursery compared to finisher) | >1 | - | <0.05 |  | F | USA | 41 |
|  | transfer through a room housing older pigs when moving a batch of pigs from the post-weaning to the fattening room | 3.3 | 1.1-9.6 | 0.03 | H1N1 | F | France | 50 |
|  | housing in gestating crates (weaner prevalence) | neg | - | 0.031* |  | F | Germany | 114 |
|  | group housing of gestating sows (sow prevalence) | pos | - | 0.025* |  | F | Germany | 114 |
|  | farrowing and nursery units placed in the same barn (weaner prevalence) | neg | - | 0.019* |  | F | Germany | 114 |
|  | suckling period ≥28 days (weaner prevalence) | neg | - | 0.011* |  | F | Germany | 114 |
|  | production system 2 vs 1 | 1.7 | 1.3-2.3 | <0.001 |  | F | USA | 76 |
| **Housing** | floor space ≤0.35m2/pig in the post-weaning pen | 2.9 | 1.2-7.0 | 0.02 | H1N2 | F | France | 50 |
|  | presence of discontinuous partitions between pens in fattening units | 5.31 | 1.59-17.70 | 0.007 |  | F | Spain | 172 |
|  | at least some pigs kept indoors | 3.59 | 1.11-11.57 | 0.03 |  | F | UK | 112 |
|  | fully slatted floor | 9.11 | - | 0.009 | H1N1 | F | Belgium | 107 |
|  | at least some pigs kept in straw yards | 0.3 | 0.11-0.82 | 0.02 |  | F | UK | 112 |
| **Environmental factors** | temperature setpoint of the heating device in the farrowing room ≤25C | 2.6 | 1.1-6.4 | 0.03 | H1N1 | F | France | 50 |
|  | temperature setpoint of the ventilation controller in the post-weaning room ≤24C | 2.6 | 1.1-6.1 | 0.03 | H1N1 | F | France | 50 |
|  | range of temperature values for the ventilation rate control in the fattening room ≤5C | 3.2 | 1.4-7.4 | <0.01 | H1N2 | F | France | 50 |
|  | temperature: 1°C increase | 1.04 | 1.01-1.07 | <0.05 |  | F | USA | 41 |
|  | wind speed: 1km/h increase | 1.24 | 1.08-1.43 | <0.05 |  | F | USA | 41 |
|  | pigs not sampled in Summer (Jul-Sep) | 2.54 | 1.09-5.95 | 0.03 |  | F | UK | 112 |
| **Biosecurity** | duration of the empty period between successive batches in the farrowing facilities <4 days after cleaning and disinfection | 2.6 | 1.1-6.3 | 0.03 | H1N2 | F | France | 50 |
|  | lack of all-in all-out in the fattening room | 2.4 | 1.0-5.8 | 0.04 | H1N2 | F | France | 50 |
|  | uncontrolled access to farm | 2.44, 3.46 | 1.01-5.87, 1.08-11.1 | 0.047, NA |  | F | Spain | 172 |
|  | lack of bird-proof nets | 2.82 | 1.08-7.40 | - |  | F | Spain | 172 |
|  | carcasses disposed by authority | 7.32 | 3.42-15.68 | 0 | H3N2 | F | Malaysia | 182 |
|  | separation of diseased pigs in sick bays | neg | - | 0.025, 0.007* |  | F | Germany | 65 |
|  | separate pen in the nursery for runting or diseased piglets (sow prevalence) | pos | - | 0.025* |  | F | Germany | 114 |
|  | housing of runting and diseased weaner pigs in a hospital pen in the farrowing unit (weaner prevalence) | pos | - | 0.007* |  | F | Germany | 114 |
|  | vaccination against PRRS | neg | - | 0.002* |  | F | Germany | 65 |
|  | vaccination against PCV2 | neg | - | 0.007* |  | F | Germany | 65 |
|  | vaccination of sows and piglets against PRRSV (sow prevalence) | neg | - | 0.002, 0.024* |  | F | Germany | 114 |
|  | vaccination of piglets against PCV2 (weaner prevalence) | neg | - | 0.001* |  | F | Germany | 114 |
|  | vaccination of gilts against SIV (weaner prevalence) | neg | - | 0.014* |  | F | Germany | 114 |
|  | vaccination of the entire sow herd against SIV (weaner prevalence) | neg | - | 0.013* |  | F | Germany | 114 |
| **Human contact** | human ILI in farm staff | 4.15 | 1.5-11.4 | 0.005 | H1N1pdm09 | F | Norway | 58 |
|  | human density | (0.0031) | - | 0.003 | H3N2 | F,S | Cambodia | 124 |
|  | employment of external swine workers | (-1.23) | - | <0.01 |  | F | Vietnam | 186 |
| **Other animal species** | contact rates between free-ranging swine and domestic ducks | pos | - | best predictor | H1N1 pdm09 | F | Cameroon | 93 |
|  | contact rates between free-ranging swine and wild Columbiformes | pos | - | best predictor | H1N1 pdm09 | F | Cameroon | 93 |
|  | contact rates between humans and ducks | pos | - | best predictor | H1N1 pdm09 | F | Cameroon | 93 |
|  | presence of avian species in farm | 4.3 | 2.10-8.75 | 0.00 | H1N1 | F | Malaysia | 182 |
|  | presence of dogs vs cats | 0.22 | 0.06-0.81 | 0.02 | H1N1 | F | Malaysia | 182 |
|  | presence of cats and dogs vs cats | 0.05 | 0.005-0.46 | 0.01 | H1N1 | F | Malaysia | 182 |
|  | no presence of cats or dogs in farm vs cats | 0.03 | 0.01-0.09 | 0.00 | H1N1 | F | Malaysia | 182 |
| **Clinical observation** | outbreak of an influenza-like disease 1–3 weeks before sampling (sow prevalence) | pos | - | 0.014* |  | F | Germany | 114 |
|  | diseased herds | 1.03 | 1.01-1.05 | -* |  | F | Germany | 121 |
|  | in-term birth of stillborn and weak born piglets (sow prevalence) | neg | - | 0.019* |  | F | Germany | 114 |
| **Geography** | Illinois vs Oklahoma | 1.9 | 1.4-2.6 | <0.001 |  | F | USA | 76 |
|  | East-Central & East regions | 0.15 &  0.12 | 0.05-0.46 & 0.03-0.53 | - |  | F | Bhutan | 118 |
| **Other** | presence of breeding show | 21.676 | 2.417-inf. | 0.005 |  | A | USA | 16 |
